# Digital Pathology Analysis Quantifies Spatial Heterogeneity of CD3, CD4, CD8, CD20, and FoxP3 Immune Markers in Triple-Negative Breast Cancer

**DOI:** 10.3389/fphys.2020.583333

**Published:** 2020-10-19

**Authors:** Haoyang Mi, Chang Gong, Jeremias Sulam, Elana J. Fertig, Alexander S. Szalay, Elizabeth M. Jaffee, Vered Stearns, Leisha A. Emens, Ashley M. Cimino-Mathews, Aleksander S. Popel

**Affiliations:** ^1^Department of Biomedical Engineering, Johns Hopkins University School of Medicine, Baltimore, MD, United States; ^2^Johns Hopkins Mathematical Institute for Data Science, Whiting School of Engineering, Johns Hopkins University, Baltimore, MD, United States; ^3^Department of Oncology, Sidney Kimmel Comprehensive Cancer Center, Johns Hopkins University, Baltimore, MD, United States; ^4^Henry A. Rowland Department of Physics and Astronomy, Krieger School of Arts and Sciences, Johns Hopkins University, Baltimore, MD, United States; ^5^Department of Computer Science, Whiting School of Engineering, Johns Hopkins University, Baltimore, MD, United States; ^6^The Bloomberg∼Kimmel Institute for Cancer Immunotherapy, Johns Hopkins School of Medicine, Baltimore, MD, United States; ^7^Department of Medicine/Hematology-Oncology, Hillman Cancer Center, University of Pittsburgh Medical Center, Pittsburgh, PA, United States; ^8^Department of Pathology, Johns Hopkins University School of Medicine, Baltimore, MD, United States

**Keywords:** digital pathology, image informatics, spatial patterns, breast cancer, tumor heterogeneity, immuno-architecture, QuPath

## Abstract

Overwhelming evidence has shown the significant role of the tumor microenvironment (TME) in governing the triple-negative breast cancer (TNBC) progression. Digital pathology can provide key information about the spatial heterogeneity within the TME using image analysis and spatial statistics. These analyses have been applied to CD8+ T cells, but quantitative analyses of other important markers and their correlations are limited. In this study, a digital pathology computational workflow is formulated for characterizing the spatial distributions of five immune markers (CD3, CD4, CD8, CD20, and FoxP3) and then the functionality is tested on whole slide images from patients with TNBC. The workflow is initiated by digital image processing to extract and colocalize immune marker-labeled cells and then convert this information to point patterns. Afterward invasive front (IF), central tumor (CT), and normal tissue (N) are characterized. For each region, we examine the intra-tumoral heterogeneity. The workflow is then repeated for all specimens to capture inter-tumoral heterogeneity. In this study, both intra- and inter-tumoral heterogeneities are observed for all five markers across all specimens. Among all regions, IF tends to have higher densities of immune cells and overall larger variations in spatial model fitting parameters and higher density in cell clusters and hotspots compared to CT and N. Results suggest a distinct role of IF in the tumor immuno-architecture. Though the sample size is limited in the study, the computational workflow could be readily reproduced and scaled due to its automatic nature. Importantly, the value of the workflow also lies in its potential to be linked to treatment outcomes and identification of predictive biomarkers for responders/non-responders, and its application to parameterization and validation of computational immuno-oncology models.

## Introduction

Triple negative breast carcinoma (TNBC) is an aggressive form of breast cancer that is negative for estrogen receptor (ER), progesterone receptor (PR), and human epidermal growth factor receptor 2 (HER-2). Treatments for TNBC have historically been confined to surgery, radiation and chemotherapies due to the lack of biologic targets which enable endocrine therapy (ER and PR) and targeted therapy (HER2) in other subgroups of breast cancer. Recent studies deciphering the role of the immune system in cancer revealed a significant effect of the tumor microenvironment (TME) in modulating the tumor progression, especially how the tumor hijacks the anti-inflammatory mechanism of inhibitory immune checkpoint molecules to develop its immune resistance and evasion capability. These studies inspired the emergence of anti-cancer immunotherapy by promoting the host anti-tumor immunity. This idea has led to an array of successful treatments against cancers. Recently, the Impassion130 study demonstrated that first-line treatment with atezolizumab and nab-paclitaxel resulted in an overall survival benefit in patients with advanced programmed death-ligand 1 (PD-L1) positive TNBC, and this is a new standard of care (Schmid et al., 2020). Meanwhile, immune checkpoint blockade also advances the treatment outcomes for other cancer types including melanoma (Long et al., 2016), non-small cell lung cancer (Melosky et al., 2020), and renal-cell carcinoma (Motzer et al., 2019). However, not all patients experience therapeutic benefit from immunotherapy. Heterogeneity within the TME may account for some of the variability in patient response to immunotherapies (Klemm and Joyce, 2015). Therefore, characterization of the tumor immuno-architecture is a critical step toward understanding the complex interplay between pro-and anti-tumor immunity.

Previous research indicates that the existence of tumor-infiltrating lymphocytes (TILs) possesses a unique predictive or prognostic value in different types of cancer (Haanen et al., 2006; Kawai et al., 2008; Suzuki et al., 2010; Ladányi, 2015; Lianyuan et al., 2018), including TNBC (Denkert et al., 2018) as the infiltrating profiles are associated with favorable patient outcomes. Specifically, high overall survival scores are often accompanied by high levels of cytotoxic CD8+ T cells, whereas forkhead box protein 3+ (FoxP3+) regulatory T cells and Type 2/Type 17 CD4+ helper T cells (Th_2_ and Th_17_ T cells) diminish this effect (Fridman et al., 2011). Therefore, monitoring the distribution of different TILs and their associations should yield insights into how cancer progresses. Hence, further studies are needed to elucidate the underlying mechanisms from the spatio-temporal perspective.

Digital pathology is an emerging discipline that allows quantitative analysis of digital images of histological specimens using computational approaches. Digitized images provide easy access for pathologists to high-resolution histological data with typically gigapixel content (Al-Janabi et al., 2012). Therefore, tissue contexts are well preserved and amenable to computer-assisted techniques for quantitative analysis of the spatial immuno-architecture. We started the development of a workflow for spatial statistical analysis in a previous study with a single immune marker for CD8+ T cells (Gong et al., 2018). The process started with image processing, during which tumor specimen images were segmented to map CD8+ T cells into a Cartesian coordinate system, then the density of point pattern within each subregion (first-order property in spatial statistics terminology) were gathered to reveal spatial variations. For each subregion, cell coordinates were converted into spatial point patterns, then Thomas cluster process was fitted to clustered patterns (assessed by complete spatial randomness test). For the entire point pattern, cell clustering morphometrics were performed. Collectively, fitted clustering parameters and morphometric measurements were harvested to characterize the immuno-architecture (Gong et al., 2018). Other investigators examined the CD8+ T cells infiltration profile by constructing profiles of cell pixel density vs. distance from the tumor boundary and then used a computational model to interpret the data (Li et al., 2019). Alternative approaches introduced the pattern of tumor cells as a reference to measure the infiltration of lymphocytes. CD8+ T cells and the tumor cells were colocalized and then a series of metrics were introduced to measure the spatial interactions such as quantifying the nearest neighbor distribution function for two different cell types using spatial G-function (Barua et al., 2018) and evaluating the spatial clustering using Morisita index and Getis-Ord hotspots analysis (Yuan, 2016). These studies adopt different metrics to interpret spatial distributions of various entities extracted from pathology data; spatial heterogeneity is a universally observed hallmark of cancer whether it is gauged in terms of density, model fitting parameters, clustering size, or infiltration level. Such variations can also be linked to treatment outcomes to better understand the effects of the TME and to assist clinicians in making more accurate diagnoses.

The integration of image processing, statistical analysis, and computational biology has already shown to be powerful in characterizing and interpreting the spatial heterogeneity in multiple tumor types including breast cancer (Brown et al., 2014; Mani et al., 2016; Altan et al., 2018; Du et al., 2019; Wong et al., 2019). Nevertheless, high dimensional quantitative measurements of the interaction between immune markers and assessment of their spatial correlations have not been conducted. Such metrics will not only provide additional layers of tumor spatial heterogeneity information valuable for patient stratification, but also allow us to better understand the mechanisms behind the formation of the patterns we observe in the TME. In this study, we propose a multi-module workflow to quantify the spatial patterns of five immune markers that control the functional status of T cells, on consecutive pathology sections: CD3, CD4, CD8, CD20, and FoxP3, and from a small patient population (*n* = 5) with TNBC to obtain statistically and pathologically meaningful results. For each patient, our workflow starts with image processing, evaluation of point patterns from three perspectives, and implementation of region-based characterization. To the best of our knowledge, our analysis evaluates the heterogeneity of TNBC in a broad immune context for the first time, therefore paving the way to the identification of reliable predictive biomarkers and the design of innovative therapies when properly correlated with clinical outcomes.

In addition, the cell densities derived from the workflow can be converted to 3D numerical densities to facilitate development and calibration of spatially resolved computational immuno-oncology models. For example, 3D densities of different cell types calculated from point patterns can be utilized to populate *in silico* computational agent-based models (ABMs) that have the potential of predicting spatio-temporal TME (Gong et al., 2017; Norton et al., 2017, 2018, 2019). Such three-dimensional ABM could be combined with Quantitative Systems Pharmacology (QSP) models for whole patient to enable mechanistic systems biology modeling of different drugs or combinations (Cheng et al., 2017; Rieger et al., 2018; Bai et al., 2019; Jafarnejad et al., 2019; Milberg et al., 2019; Wang et al., 2019, 2020; Sové et al., 2020).

## Materials and Methods

### Pathology Specimen Materials and Methods

This study was approved by the Institutional Review Board of the Johns Hopkins Medical Institutions. Digitally scanned slides from a subset of previously described primary breast tumors were evaluated (Cimino-Mathews et al., 2016). Briefly, formalin fixed, paraffin embedded blocks from surgically resected primary breast carcinomas with no prior neoadjuvant chemotherapy were randomly selected from the pathology archives at Johns Hopkins Medical Institutions (associated response data to treatment not available). TNBC was defined as negative for ER, PR, and the HER-2. Consecutive sections (approximately 5 μm each) from whole tumor were individually stained for CD3 (mouse monoclonal, clone PS1, catalog no. ORG-8982; Leica Microsystems, Bannockburn, IL, United States), CD4 (rabbit monoclonal, clone Sp35, catalog no. 790-4423; Ventana Medical Systems), CD8 (mouse monoclonal, clone C8/C8144B, catalog no. 760-4250; Cell Marque, Rocklin, CA, United States), CD20 (monoclonal, clone MS/L26, catalog no. 760-2531; Ventana Medical Systems, Tucson, AZ, United States), and FoxP3 (mouse monoclonal, clone 236A/E7, catalog no. 14-4777-80, dilution 1:50; eBioscience; San Diego, CA, United States). Immunohistochemically labeled slides were scanned at 20× objective (0.49 microns/pixel) using the Aperio Scanscope AT (Aperio/Leica Biosystems, Vista, CA, United States). Five (5) cases of TNBC were selected for this current study based on intact tissue integrity on the scanned images (i.e., complete cross sections and lack of tissue folds). To simplify the analysis, two tumor islands in Case 1 are split into Case 1A (upper left island) and Case 1B (lower right island). Supplementary Figure S1 shows a representative biomarker panels across Cases 1–5.

### Computational Methods

**FIGURE 1:**
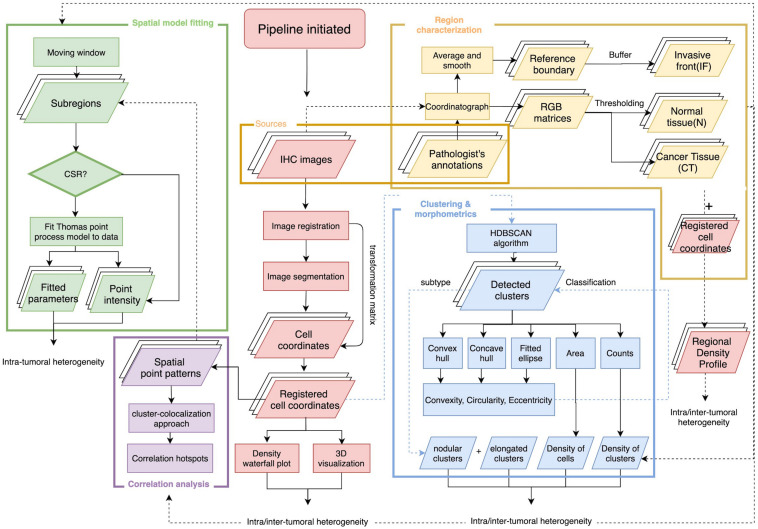
Overall workflow of spatial pattern quantification for immune markers. The workflow is initiated by two steps, first the image processing for IHC slides to extract coordinates of immune marker-labeled cells; second, the tissue type regions are characterized based on pathologist’s annotations of the tumor boundary and original IHC images. The results from these two steps construct regional density profiles. The point patterns are fed to all remaining submodules to quantify intra- and inter-tumoral heterogeneity. In the spatial model-fitting submodule, point patterns within subregions are tested for CSR, and the Thomas model is fitted to data if test is rejected. In the clustering and morphometric submodule, point clusters are detected and multiple shape descriptors are calculated for each cluster. In the correlation analysis submodule, a cluster-colocalization based method gauges the spatial distributions near each point to identify correlation hotspots, where highly correlated immune marker pairs are engaged in. For each slide, the collective results from the aforementioned metrics capture intra-tumoral heterogeneity and the analyses repeated across all cases capture inter-tumoral heterogeneity.

The overall workflow includes a central module and four submodules (Figure 1). First, the stained (positive) cell nuclei are detected and the tissue annotation module is launched to identify regions of normal tissue (N), central tumor (CT), and invasive front (IF). In this module, image registration is performed for each case on the five slides with different labels, and raw coordinates obtained from image segmentation are mapped to the same reference coordinate system using the transformation matrix. Cell densities are the output of this step, and therefore can be directly visualized using 3D and waterfall graphical representations to visualize intra- and inter-tumor heterogeneity. Also, cell density vs. distance profiles can be constructed. In the spatial point model-fitting module, for each slide, the full point pattern is divided into smaller patches with overlaps, and the Thomas point process model is then fitted based on subregion data if complete spatial randomness (CSR) hypothesis is rejected for this subregion (Baddeley et al., 2015). In the clustering and morphometrics module, for the full point pattern of each slide, the cell clusters are detected using a hierarchical clustering algorithm (Tang et al., 2016). For each detected cluster, morphometrics including convexity, circularity, and eccentricity are calculated and recorded. In the correlation analysis module, a clustering and degree of colocalization-based method is applied to quantify the correlations between immune marker pairs. All spatial statistical measurements used in this study have shown promising application values in the context of digital pathology. Results from these metrics are classified based on tissue type regions and then used for statistical comparison to reveal intra-tumoral heterogeneity. Such workflow is repeated for all cases, thus capturing inter-tumoral heterogeneity.

#### Image Processing

##### Cell nucleus segmentation and coordinate extraction from IHC slides

**FIGURE 2:**
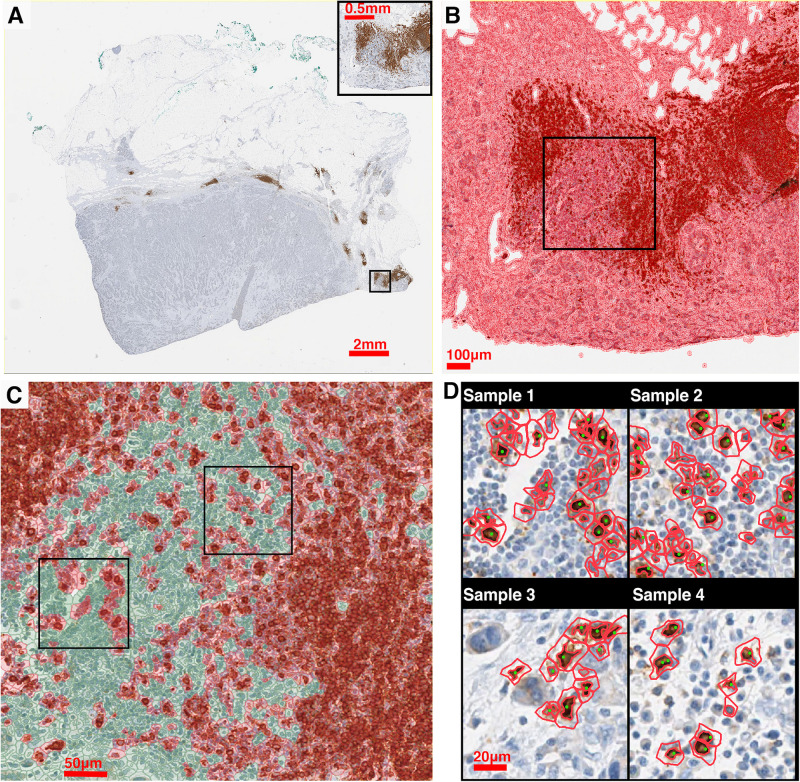
IHC image segmentation, classification using QuPath and performance validation. **(A)** Original IHC image (Case 5, CD20). **(B)** Nucleus detection results of the subregion marked in panel **(A)**. In this process, nucleus boundaries are detected (inner red contour) and then expanded outward for 7.5 μm (outer red contour) to form a pseudo cell. Then morphology and intensity features of the cell objects are fed to the classifier to identify positive cells. **(C)** Classification results of the subregion marked in panel **(C)** using the corresponding classifier. Green: negative (non-stained) cells. Red: positive (stained) cells. **(D)** Results of manual detection (green dots) and algorithmic detection (red outlines) are mapped together to evaluate the performance. 20 subregions are randomly sampled across the slide. Two subregions marked in panel **(C)** are shown as examples.

Stained (positive) nucleus segmentation is performed using software platform QuPath (v0.2.0-m10) (Bankhead et al., 2017). QuPath is selected for this study because it is a flexible open-source software with well-managed version control and technical support, and it is capable of a wide range of digital pathology analyses. As the IHC staining may vary both between and within each case, the image processing workflow is initiated by manual correction to the stain estimates for each whole slide image using the ‘estimate stain vectors’ function. Nucleus detection is then carried out using an unsupervised watershed algorithm with custom parameters tuned on a validation set of WSIs from Case 1, 2, and 3. This built-in algorithm has been implemented by a wide range of peer-reviewed studies (Bankhead et al., 2018; Zhang et al., 2018; Acs et al., 2019a,b; Ferré et al., 2019; Habets et al., 2019; Kather et al., 2019; Santiago et al., 2019; Berben et al., 2020; Blagih et al., 2020; Tsakiroglou et al., 2020). Importantly, the performance was found equivalent to commercial software and pathologists’ manual annotations (Bankhead et al., 2018; Acs et al., 2019b; Berben et al., 2020). While the nuclei are identified, centroid coordinates are recorded to represent the cells’ location. Afterward, pseudo cell objects are formed by expanding the nuclei boundaries for 7.5 μm and then a list of features is calculated based on intensity and morphometry measurements. For each IHC biomarker, 25 regions of interests (ROIs) are randomly selected for Cases 1–3 (75 in total) and a Random Tree classifier (Breiman, 2001) is trained using the aforementioned features by annotating regions in a subset of subregions. The classification results are updated in the form of color marks when each annotation is drawn. The classifier is then validated on the remaining WSIs to ensure the robustness. A low-resolution image in the testing set is shown in Figure 2A. Exemplar segmentation and classification results are shown in Figures 2B,C. Segmentation and classification settings are shown in Supplementary Material and Supplementary Tables S1, S2.

To evaluate the performance of the image segmentation algorithm, 20 subregions are sampled from each slide for each case using the random sampling method. Figure 2D shows four exemplar subregions for performance evaluation, where red outlines indicate QuPath segmentation results and green dots indicate manual approach. For each subregion, we also manually detect labeled cells, and then measure the sensitivity (recall) and precision of our algorithm. Results indicate that there is a strong correlation between manual and automatic approaches (Spearman’s correlation coefficient ρ = 0.978). Details of the evaluation of QuPath can be found in the Supplementary Material.

##### Registration and coordinate transformation of IHC slides

**FIGURE 3:**
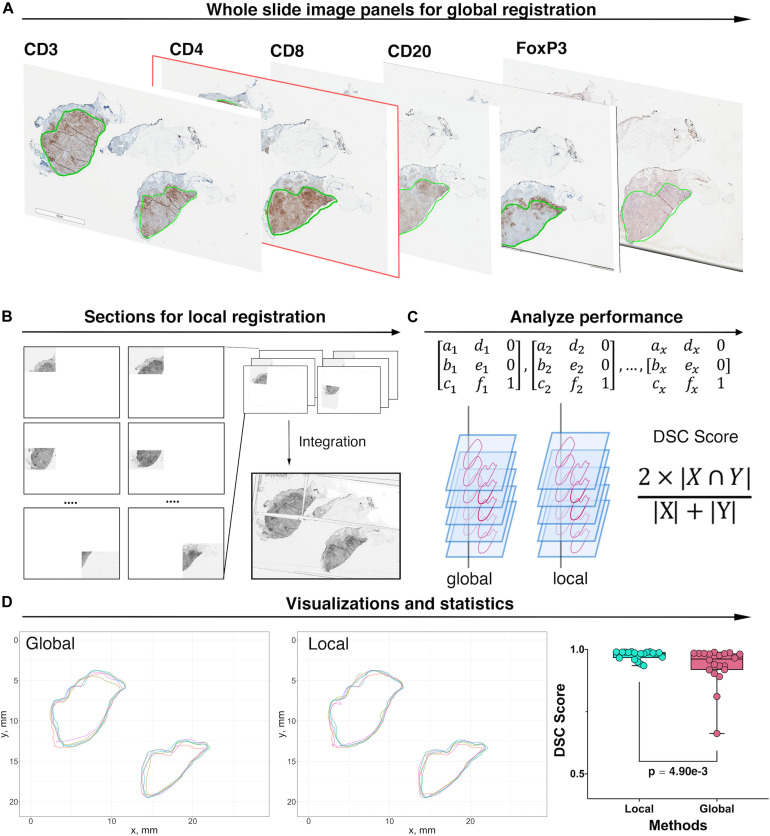
Image registration and performance evaluation workflow, Case 1 is taken as an example for illustration. **(A)** Whole slide image panels with pathologist’s annotations (green outlines) for Case 1. In this study, CD4+ slide is selected as the reference. Global registration is first applied to all registration pairs. The performance for each pair is then evaluated manually and prepared for local registration if necessary. **(B)** Poorly registered slides are subject to local registration. Slides and references are segmented into multiple subregions and using software Icy to perform local pairwise registration. **(C)** Transformation matrices obtained from both local and global registrations are applied to tissue contours. For each method, the DSC is then computed between registered contours and the contours of the reference slide. **(D)** Registered contours from two methods and reference contours are mapped to the same coordinates. DSC is computed by calculating their respective and intersection areas. The Wilcoxon rank-sum test is performed when DSCs for all 20 registrations pairs are collected. The result showed that the local registration performs significantly better than global registration (Wilcoxon rank-sum ***p*** = **4.90e-3**).

The pathology images available for this study were single label IHC slides produced with consecutive sections from each tumor excision. In this process, z-axis difference for each section, location and rotation when placed onto slides, as well as possible folding of tissue during preparation, all contributed to discrepancies between coordinate systems of each slide from the same patient. These discrepancies are minimized by image registration. As the cutting sequence of these immune marker slides was unknown, all slides are treated equally and the CD4+ slide is selected as the reference for all cases. Global automatic registration by Matlab application “Registration Estimator” is first performed for all pairs (Figure 3A). The registration accuracies are manually assessed based on tissue overlap level: among 20 registration pairs, we find that global registration produces high accuracy for five pairs; for the remaining, the local registration is required, which is performed using software Icy (De Chaumont et al., 2012) (Figure 3B). Both global and local registrations generate transformation matrices for the corresponding regions. These matrices can be used to estimate registration accuracy. First, global registrations are performed for all pairs as the baseline. Next, transformation matrices generated from global and local registrations are used to register tissue contours, separately. Dice Similarity Coefficients (DSCs) (Guy et al., 2019) are calculated, respectively, and cumulative results are collected (Figure 3C). Finally, we compared the registration accuracy by performing the Wilcoxon rank-sum test between global and local DSC groups (Figure 3D) and observed a significant improvement (***p*** = **4.90e-3**). Results also show that the average global registration DSC scores for those five pairs (0.916) are very similar to the average local registration DSC scores (0.917). Technical details on global and local registrations and performance evaluation can be found in the Supplementary Material.

#### Measuring Intra- and Inter-tumoral Heterogeneity

##### Region characterization based on pathologist’s annotations

**FIGURE 4:**
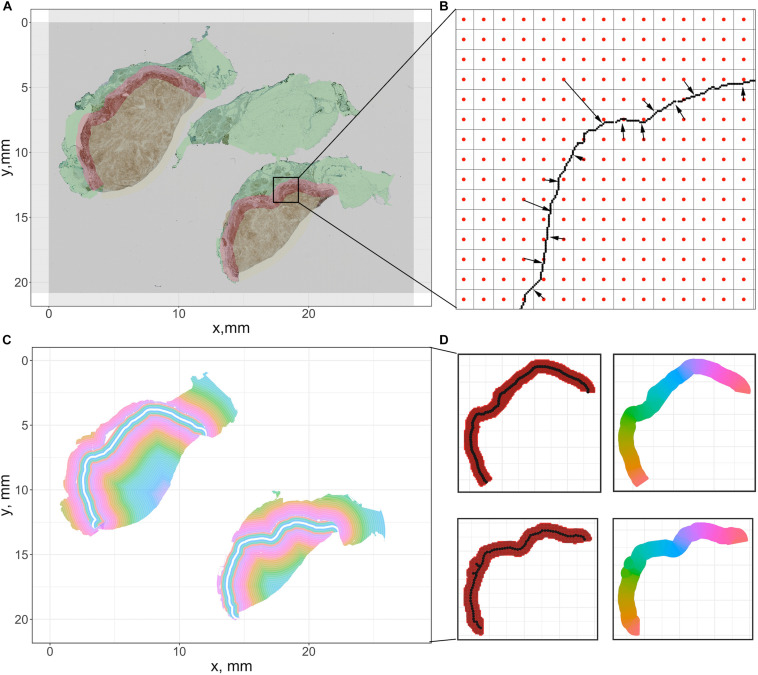
Summary of the pixel-distance based method to generate sections for cell density-distance profiles. We use Cases 1A and 1B here for illustration. **(A)** For the downsampled image, we generate a grid whose size is equal to the number of pixels. Then the center coordinate of each square is recorded to represent the pixel. **(B)** For all pixels within tissues, we calculated the distance toward the tumor boundary of the corresponding case. **(C)** All pixels are grouped based on their distances to generate equidistant sections. **(D)** To segment the invasive front, multiple equidistant points are sampled along the boundary, and each pixel within the IF is assigned to their nearest sample point. Then all pixels associated with each sample points form a polygon to calculate density. The color codes in the right panels indicate different sections.

For each case, the breast cancer pathologist (AMC-M) annotated (outlined) the tumor boundary, which was considered the ‘ground truth’ for the present analysis. Green contours in Figure 4A and Supplementary Figure S2 indicate the annotations for Case 1 and Cases 2–5. Annotations are converted into coordinate sets and then registered to the reference slide (CD4+ slide) using the transformation matrices. Each annotation is a closed curve so that the corresponding coordinate set can form a polygon. Next, we create a score map by overlaying the five coregistered polygons (from five slides, one for each immune marker) and recording the number of polygons each pixel of the WSI resides within. Now that each pixel is assigned a score, we apply a smoothing filter to the score map and threshold all pixels with scores exceeding 2.5 to determine the consensus tumor boundary. Finally, we obtain a dense region, the contour of which functions as the averaged boundary between normal tissue [sometimes referred to as stroma (Tyekucheva et al., 2017)] and CT. Next, we buffered the boundary with 0.5 mm inward and outward to create the IF, which separates the normal tissue (N) and CT with a band of 1 mm (Pages et al., 2009; Halama et al., 2011; Hendry et al., 2017). Note that in some studies the region is considered 0.5 mm wide (Gong et al., 2018; Li et al., 2019); we will show below the quantitative implications of either assumption. Afterward, we remove those pixels that fall within the IF from the tumor mask, thus the contour of remaining points gives the outline of CT. Similarly, to extract the normal tissue, we computed the mean value of the RGB channels for each pixel. To exclude background and noise, we set a customized threshold to rule out high-intensity pixels. The remaining pixels contain complete WSI foreground information, and the normal region can be easily extracted when IF and CT pixels are removed. For all pixels associated with each region, we obtain the outline to form a polygon to represent the region. In this step, specimen images and annotations from pathologist are the only inputs. Pixel coordinate maps are generated using python. All subsequent computations are performed using R: the point-in-polygon test is performed using ‘point.in.polygon’ function from R package ‘sp’ (Pebesma and Bivand, 2005); the outlines are generated using ‘concaveman’ function from R package ‘concavemann’ (Gombin et al., 2017).

##### Whole slide image partitioning and extraction of first-order properties

We partition the WSI into subregions for local spatial analysis using a moving rectangular window with edge lengths of *x*_*window*_ and *y*_*window*_, which traverses the WSI with step size of *x*_*step*_ and *y*_*step*_. The window size should be large enough to capture local density variations, and sufficiently small to have multiple subregions and stationary underlying point pattern processes. Based on these considerations, we performed fractal analysis (see Supplementary Material and Supplementary Figure S3) and determined the window lengths and step size as *x*_*window*_ = *y*_*window*_ = 0.4 mm and *x*_*step*_ = *y*_*step*_ = 0.2 mm (complete discussion is in Supplementary Material). In this study, we define all individual rectangular areas that the moving window has scanned as subregions. As the window is moving through the whole slides, first-order properties such as number and density of points are recorded for subsequent visualization and local statistical analysis.

##### Measuring the heterogeneity with spatial entropy measurement

A form of Shannon’s entropy (Claramunt, 2005) uses the entropy as a measure of diversity of density of multiple point species in space. This modified version incorporates the factor of distance. Assuming that the increase of distance between same type of points and the decrease of distance between different types of points will result in the increase of entropy, this spatial entropy is defined as:

(1) $$H_{\mathrm{SC}}=-\sum_{i=1}^{n}\frac{d_{i}^{\mathrm{int}}}{d_{i}^{\mathrm{ext}}}\,p_{i}\,\log_{2}p_{i}$$

where $d_{i}^{i\,n\,t}$ is the average Euclidean distance between all points of type *i*; $d_{i}^{e\,x\,t}$ is the average Euclidean distance between all points of type *i* and the points of other types; *p*_i_ is the percentage of type *i* within the subregion.

For each case, we first map registered and reference point patterns into a Cartesian coordinate system. Next, we calculate the *H*_*sc*_ for the multi-type point pattern within subregions. Collective results are then classified based on tissue type associated with their locations. Then we examine intra- and inter-tumoral heterogeneity of spatial entropy in each tissue type by showing their distributions with a series of probability density functions.

##### Constructing cell density versus distance profile for whole slide image

To compute cell density-distance profiles, infiltration intensities are quantified as the immune-marker labeled cell densities at the corresponding distance from consensus tumor boundary obtained in the previous step. To generate the density-distance profile, we utilized pixel-distance based algorithm to segment the whole tissue into multiple equal-width band sections: all foreground pixels are classified according to their locations, denoted as CT-, IF-, and N-pixels; for both CT- and IF-pixels, the distance toward the IF inner (adjacent to CT) boundary are computed; for each N-pixel, the distance toward the IF outer (adjacent to normal tissue) boundary are computed; next we group the pixels into multiple intervals with the interval length of 150 μm (first interval: 0–150 μm; second interval: 150–300 μm; …) based on their distance value; then we extract the shape outline for each group by computing the concave hull; consequently each group comprises a polygon with 150 μm width. Minor adjustments are needed to ensure all points within each shape outline are properly arranged so that they can be connected in clockwise or counterclockwise direction, if necessary.

To ensure the density-distance profile proceeds along the direction of immune infiltration, first we assign index 0 to the central band polygon of the IF as the reference polygon. Then we render negative indexes, decreasing from 0 until we reach the most distant band within the normal tissue, and render positive indexes, increasing from 0 until we reach the most distant polygon within the tumor. With all groups are arranged in a consecutive numerical order that expands from the edge of normal tissue toward the CT, the cell density is generated by calculating the area and counting the cells inside. The described methods are summarized in Figures 4A–C.

For all aforementioned calculations, the concave hull is computed using a function from R package ‘concaveman’; the pixel distance and polygon areas are computed using the function ‘gDistance’ and ‘gArea’ from R package ‘rgeos’ (Bivand and Rundel, 2017).

##### Constructing cell density versus distance profile within invasive front

The IF is segmented into sections along its own direction: the length of central reference line is first calculated; then multiple equispaced points along the line are sampled based on a given interval. A Voronoi tessellation was created within the IF region based on Euclidian distance to each chosen point. The area of each resulting polygon and the number of cells it encloses are computed to determine cell density, and all polygons are indexed in a numerical order starting from 1 with the left-most point, in a clockwise direction. The methods are summarized in Figure 4D. The length of the reference line is calculated using the function ‘lineLength’ from R package ‘SDraw’ (McDonald, 2016); the length of interval is set to be 0.2 mm; the points are sampled using the function “spsample” from R package ‘sp.’

##### Constructing 95% confidence interval (CI) along with the cell density profile

Depending on the shape of whole slide images, polygons at a certain distance may be small in size, which could result in inaccurate estimation of the average cell density of that distance. Therefore, to test the reliability of all average cell density point estimates along the 2D projection, we construct a 95% CI along the profile. For better accuracy, we assume the variance of density between each window within one distance polygon band is equal to the variance of the entire region with the same tissue type as the polygon of interest, which is denoted as σ. Hence the confidence interval can be computed according to the formula (Efron, 1981):

(2) $$D\pm T_{c}\cdot \frac{\sigma }{\sqrt{n}}$$

where *D* is the density of cells within a polygon, *T*_c_ is the critical *t*-value. In this study *T*_c_ is defined as 1.96 (95% confidence level); n is the number of samples, and is calculated according to the equation:

(3) $$n=\frac{A}{s_{l}\times s_{w}}$$

where *A* is the area of the polygon; *s*_*l*_ and *s*_*w*_ are the length and width of the window. In this study, window size is defined as *s*_*l*_ = *s*_*w*_ = 0.4 mm.

Confidence intervals are calculated along with the density profile; however, when visualizing the data, we truncated the portion below zero, and use 80% of the density as the threshold to filter out locations at which the density estimates are not reliable as the mean of the region (labeled as red dots).

##### Constructing the three-dimensional immune landscape

For each slide, the recorded density mapped to original locations to construct the landscape. The entire landscape is then characterized to reflect region-specific information (N, IF, and CT). The landscape data visualization is implemented using software Blender 2.80 (Hess, 2007).

##### Measuring the heterogeneity from spatial point pattern process model fitting results

In our study, the local point pattern is defined as the point pattern of the immune marker within a given subregion. For each captured local point pattern, Complete Spatial Randomness (CSR) is tested using the Clark-Evans test with the null hypothesis being a uniform Poisson process (one-tailed, *H*_*A*_: clustered distribution, significance level α = 0.05) (Baddeley et al., 2015). If the pattern failed to pass the CSR test, we fit a Thomas point process model to the local point pattern and record fitted parameters. The model assumes cluster patterns are generated in two steps: in the first step, a pattern of parent points within the window is generated according to a homogeneous Poisson process given the intensity κ; in the second step, a random number of offspring points is generated, so that the number of offspring points that belong to any parent point also follows Poisson distribution with intensity μ, at the same time the location follows isotropic Gaussian distribution with standard deviation σ. The theoretical Ripley’s *K* function of the Thomas process is:

(4) $$K\,\left(r\right)=\pi \,r^{2}+\frac{1}{\kappa }\,\left(1-e^{-\frac{r^{2}}{4\,\sigma^{2}}}\right)$$

where *r* is the distance of a sample random point of the point pattern within which the function is evaluated. For each sub-region, the fitted parameters κ, μ, and σ are biologically interpreted as features of the clustering pattern of immune marker-labeled cells. κ stands for the number of labeled cell clusters per unit area; μ is the number of labeled cells per cluster. We further use the distance of the cell toward the cluster center to quantify the internal cell distribution of each cluster (Thomas, 1949; Waagepetersen, 2007; Tanaka et al., 2008). We observe that the components of distance vector are normally distributed and independent since each point in the generated clustered pattern is produced from an isotropic Gaussian process so that the collective distance profile within each cluster follows a Rayleigh distribution with a probability density function:

(5) $$G\,\left(r;\sigma \right)=\frac{r}{\sigma^{2}}\,e^{-\frac{r^{2}}{2\,\sigma^{2}}}$$

The average moment can be calculated as

(6) $$\mu \,\left(r\right)=\sigma \,\sqrt{\pi /2}$$

and the radius of the circle where 95% of the cells would fall in is calculated as:

(7) $$Q\,\left(F,\sigma \right)=\sigma \,\sqrt{-2\,\ln\,\left(1-F/100\right)}$$

where *F* = 95. The CSR testing is performed using functions “clarkevans.test” and “kppm” from R package “spatstat,” with parameters “clustered” and “Thomas,” respectively (Baddeley et al., 2015).

For each slide, we measure and compare spatial statistics between different regions (intra-tumoral) and between different cases (inter-tumoral). We use the quartile coefficient of dispersion (QCoD) and the coefficient of variation (CoV) to assess the variability of the metrics (density and spatial model fitting parameters) within one slide. Furthermore, the median value of each metric is used to represent the corresponding case for the case-wise comparison. QCoD and CoV are computed using the following formulas:

(8) $$\mathrm{QCoD}=\frac{Q_{3}-Q_{1}}{Q_{3}+Q_{1}}$$

(9) $$\mathrm{CoV}=\frac{\sigma }{\mu }$$

where *Q*_*1*_, *Q*_*3*_, *μ*, and σ are the first quartile, third quartile, standard deviation, and mean of each metric.

##### Measuring the heterogeneity from clustering and morphometric analysis

Immune contexture heterogeneity in the TME holds a significant value to the study of the anti-tumor immune response (Beck et al., 2011; Schwen et al., 2018). Therefore, we performed immune cell cluster analysis to assess the intra- and inter-tumoral heterogeneity. We first identify clusters from the global point patterns using an adjusted version of the clustering algorithm Hierarchical DBSCAN (HDBSCAN) from R package ‘largeVis’ (Tang et al., 2016). This method generates the cluster hierarchy based on density-adjusted distance connectivity, and parent and child cluster stabilities are compared to extract clusters. The algorithm arguments are defined as minPts (minimum cells per cluster) = 30, and *K* (the number of cells in the core neighborhood) = 4. Next, morphological analysis is performed on each previously identified cluster by calculating shape descriptors. To describe the structure of clusters, we introduced α-shape, which envelops a set of points by point pairwise segments that could be regarded as a chord of a circle with a given radius α. To identify the α-shape which exactly harbors all points within the given region, the value of α is increased from 10 μm until the polygon reaches the ideal size. α-shapes are computed using the function “ashape” from R package ‘alphahull’ (Rodríguez Casal and Pateiro López, 2010). Then the morphometrics for each cluster are calculated for the following measurements:

**Convexity:** Measures the degree of the object. Convexity is mathematically defined as:

(10) $$f_{\mathrm{convex}}=\frac{A_{\alpha }}{A_{\mathrm{convex}}}$$

where *A*_α_is the area of the *α*-shape and *A*_*convex*_is the area of the convex hull, generated upon the same dataset.

**Circularity:** Measures the roundness of the object. Circularity is mathematically defined as:

(11) $$f_{\mathrm{circularity}}=\frac{4\cdot \pi \cdot A_{\alpha }}{P_{\alpha }^{2}}$$

where *P*_α_ is the perimeter of the *α*-shape.

**Eccentricity:** Measures the degree of deviation of the object from being circular. To generate the ellipse, we first assume the points within each cluster follow chi-squared distribution Q ∼ *x*^2^(*k*). Then the eigenvectors can be calculated from the covariance matrix, which indicates orientations. Now the semi-major and -minor axis lengths can be computed as:

(12) $$a=\sqrt{\lambda_{1}\,X_{2}^{2}\,\left(0.95\right)},b=\sqrt{\lambda_{2}\,X_{2}^{2}\,\left(0.95\right)}$$

where λ_*1*_ and λ_*2*_ are eigenvalues of the covariance matrix. By this definition, the ellipse is represented as the contour where 95% of points were covered. Thus, the eccentricity is computed as:

(13) $$e=\sqrt{1-\frac{b^{2}}{a^{2}}}=\sqrt{1-\frac{\lambda_{2}}{\lambda_{1}}}$$

#### Correlating the Spatial Patterns of Different Immune Markers

The metrics above all referred to spatial distributions of a single marker. However, it is of interest to know if the distributions of cells with different markers are correlated. For example, CD8+ cells generally inhibit tumor growth whereas FoxP3+ cells generally promote tumor growth; if they are colocalized their effects might cancel each other. Such assumption simplifies the definition of T cell lineages due to the limitations in materials and the scale of biomarker panel. In this study, we implement a variation of the Clus-DoC (clustering-degree of colocalization) approach to analyze spatial correlation between different immune cell labels (Pageon et al., 2016). We focused on the correlation between three pairs of the spatial patterns: CD3+/CD8+, CD4+/FoxP3+, and CD8+/FoxP3+ as representations for anti-tumor immunity regions, immunosuppression regions, and immune-crosstalk regions. With each pair of full point patterns, for both channels, a DoC score is assigned to each point. This step requires the comparison of the spatial distribution of all the neighboring points from both channels for every single point. Centered at each point of type A, circles with increasing radius are formed to compute the associated density gradients of points from both channels. Then for each point of type A, the correlations between the density gradients between both channels are measured by Spearman’s rank coefficient ρ_AB_. Next, each coefficient ρ_AB_ is converted to a DoC score by normalization using the equation:

(14) $${\mathrm{DoC}}_{A}=\rho_{\mathrm{AB}}\cdot {\text{e}}^{-\left(\frac{{\text{N}}_{\text{AB}}}{{\text{R}}_{\text{max}}}\right)}$$

where *N*_AB_ is the distance of the current point of type A to the nearest neighbor of type B, *R*_max_ is the maximum search radius. Thus, the DoC score is bounded within [−1, 1], where 1 indicates a strong correlation (colocalization) and −1 indicates anti-correlation (segregation). These calculations are performed for both channels and DoC scores are then used to identify correlated points. To select a proper DoC threshold, we create a synthetic point pattern by shifting the full point patterns of a given slide (we use CD8+ slide from Case 2 in our study, but it can be any WSI within the study cohort) to a given direction by a minor distance to simulate a well-localized pattern pair. For simplicity, we unify the shift directions for all points to left and with a distance to 10 μm plus uncertainty caused by the registration error. The averaged DoC score is then selected as the threshold. Spatially, points with high DoC scores (correlated) are close to other points of both channels, whereas points with low DoC scores (non-correlated) are not close to points of at least one channel.

In the second step, the threshold is used to select highly correlated points for each channel. Points from both channels are then mapped to the same coordinate system. Next, we use the density-based clustering algorithm described in the morphometric module to detect clusters that contain points of both types A and B. Such clusters highlight regions with strong mutual interactions of immune markers. The search of neighbors for each point is calculated and accelerated using the C++ implemented k-dimensional tree search algorithm in Python library ‘SciPy’ (Virtanen et al., 2020); the distance of a point to its nearest neighbor is calculated using function ‘nncross’ in R package ‘spatstat’ (Baddeley et al., 2015); the density-based clustering is performed using function HDBSCAN in R package ‘largeVis,’ with arguments *K* = 4, and minPts = 30.

## Results

### Assessing Intra- and Inter-tumoral Heterogeneity With Multiple Metrics

#### Immune Cell Density Distribution and Infiltration Profiles

**FIGURE 5:**
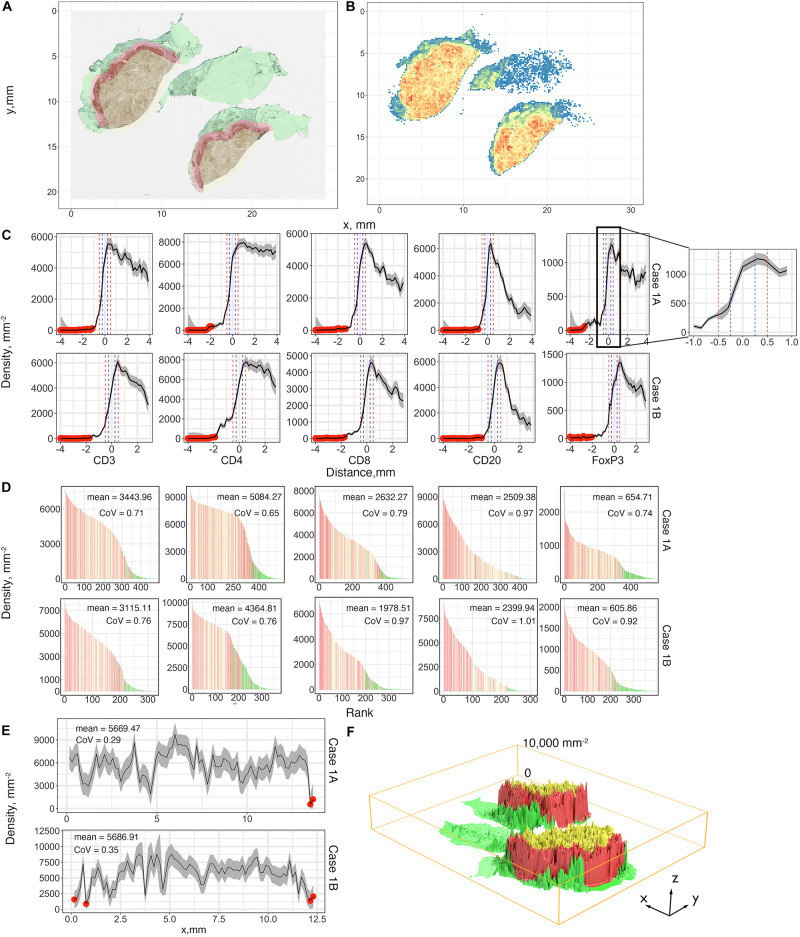
First-order variables statistics summary, Case 1, AB, is taken as an example for illustration. **(A)** Region characterization for WSI. Green: normal tissue (N); Red: invasive front (IF); Yellow: central tumor (CT). **(B)** CD4+ cell density visualized using heatmap with bin size = 0.15 mm. Color code: blue to red corresponds to low to high. **(C)** Cell density-distance profiles with a pop-up window for Cases 1A and 1B. Whole tissues are segmented into equidistant sections. Densities of different immune markers are calculated for each section and mapped with their distances to the invasive boundary, respectively. 95% confidence intervals are calculated upon the profile, and we use 80% of the density as the threshold to label those unreliable locations (red dots). Two definitions of IF are introduced here and are indicated as vertical lines, blue: width of 0.5 mm; red: width of 1 mm. **(D)** Densities of subregions are visualized using waterfall plots. For each slide, the densities are shown as bar heights, which are ranked from highest to lowest with colors corresponding to their locations. Color codes are consistent with **(A)**. **(E)** The invasive front with thickness of 1 mm is sectioned along its horizontal direction, and the same process is repeated to construct the cell density-distance profile. **(F)** 3D visualization for the density of each subregion with location labels. Color codes are consistent with **(A)**. See also Supplementary Material (Supplementary Figures S6, S7) for additional visualizations for Cases 2–5.

A summary of first-order properties analysis is shown in Figure 5. Cases 1A and 1B are used here for illustration; both parts of the tumor are present on the same slide but are separated. For each slide, the region annotations (Figure 5A) and coordinates extraction (Figure 5B) are performed first to characterize the spatial distribution. Figure 5C shows profiles of cell density vs. distance from the boundary in mm for each case for the five labels. These are the immune-infiltration profiles for different cell types. Two definitions of the IF are introduced here, with a width equals 0.5 mm (blue vertical dashed lines) and 1 mm (red vertical dashed lines) in accordance with pathologists’ convention. 95% confidence intervals are depicted in gray bands. Most of the sections consist of sufficient 0.15 × 0.15 mm windows to estimate CI; however, near the edges of the slide, in normal tissue (N) and CT, the areas may be small resulting in wide confidence intervals; in these cases we first exclude all the small regions (area < 1.1 mm^2^) from analysis and then we manually set a threshold with ±80% of real density to label the remaining unreliable sections (red dots). Importantly, we observe that the immune-infiltration profiles are unbiased regardless of how IF is defined. The maxima for the different immune cells are shifted from the boundary toward the CT, but still are within the IF. In other words, the cell densities peak around the 0.2–0.35 mm band and then drop gradually toward the innermost of the CT. To further corroborate that such infiltration profiles are caused by tumor heterogeneity, we compare the actual cell distribution pattern to a binomial distribution pattern by quadrat test. Theoretically, if a point set is randomly generated over a region which consists of multiple sections, then the expected number of points each section harbored can be calculated as the total number of points multiplied by the probability a point happened to be in this section. Therefore, for each WSI, we harvest the actual cell counts (frequencies) and theoretical frequencies in each section and performed chi-square independent test. Results show that the null hypothesis (two types of observation are independent) is rejected by all trials, suggesting that the actual cell distribution pattern is not a realization of randomness, rather it is driven by heterogeneity (Supplementary Figure S4).

Waterfall plots are frequently used in other studies to present results of clinical trials, when patients’ responses are ranked from best to worse, using tumor size as a metric, each patient is represented as a bar in the plot. In this case we use waterfall plot to rank cell densities from largest to smallest, from 0.4 × 0.4 mm windows throughout the tissue, for each label with colors corresponding to N (green), IF (red) and CT (yellow). The results are shown in Figure 5D. Waterfall plots indicate that CT and IF tend to have higher cell densities whereas fewer cells tend to accumulate in N, as the left-hand side of the chart contains more red (IF) bars and right-hand side have more green bars; the plots illustrate a high degree of heterogeneity as bars of different color are interspersed throughout the tissue. As our study focuses on heterogeneity of tumor characteristics, it is important to assess the level of heterogeneity within the IF. Figure 5E depicts the cell density distribution of CD4+ T cell plotted as a function of the distance along the middle of the IF; the densities are averaged over the width of the IF of 1 mm. Clearly, the spatial heterogeneity is present not only between different tumor regions, but also within the IF. Again, quadrat test is performed to assess the distribution pattern across all tessellations within each IF. Similarly, tests reject the null hypothesis so that the tumor heterogeneity is also the key factor that dominate the infiltration profiles in IF (Supplementary Figure S5). We then depict the immune landscape by visualizing the cell distribution in 3D (Figure 5F). For each slide, we map recorded subregion densities to their corresponding locations and generate surface plot with density represented by magnitude and region categories represented by different colors. 3D landscape representation directly depicts the regional density variations. We repeat the analysis for Cases 2–5 for all five labels and the results are presented in Supplementary Figure S6 (infiltration profiles), Supplementary Figure S7 (waterfall plots), Supplementary Figure S8 (infiltration profiles in IF), and Supplementary Figures S9, S10 (3D plots). Note that the cell density level in Cases 1A and 1B are significantly higher compared to other cases, which may reflect an efficient immune infiltration.

#### Spatial Entropy of Multitype Point Patterns

**FIGURE 6:**
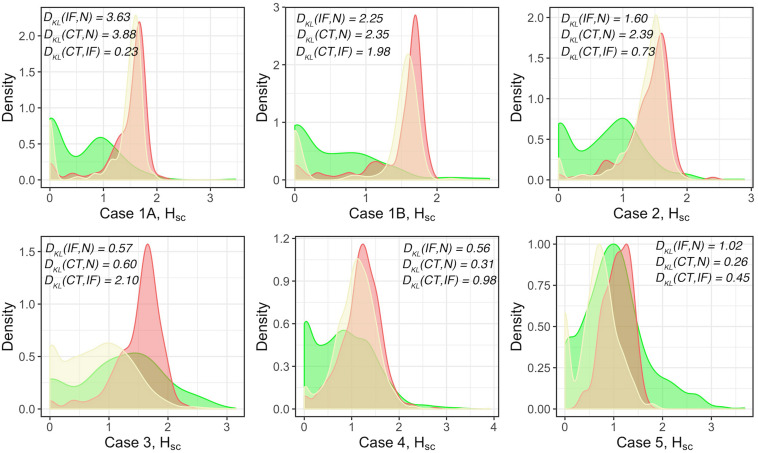
Modified Shannon’s method to quantify the spatial entropy of multitype point patterns, denoted as H_SC_. Green: normal tissue (N); Red: invasive front (IF); Yellow: central front (CT). For each case, full point patterns for each label are mapped to the same coordinate system and the H_SC_ scores are measured and presented as the PDFs. In general, the higher the *H*_SC_ score is, the more disorder/heterogeneity the subregion contains. Strong heterogeneity is observed in IF as indicated by the clustered *H*_SC_ scores across cases.

The results above visualize the heterogeneity of cell density distributions within and between the different regions of the specimens. The coefficient of variation CoV is one metric that characterizes the level of heterogeneity. We will also use a spatially adjusted Shannon’s entropy as a formal metric of spatial heterogeneity. For each WSI, the point pattern for each subregion is mapped to a Cartesian coordinate system to form a series of multitype point patterns. For each multitype point pattern, the modified Shannon’s entropy is calculated, and collective statistics are presented using probability density functions (PDFs, Figure 6). The results show clear clustering patterns of the entropy scores around 1.5 over H_SC_ measurement spaces in IF across all cases. The results indicate that regions with higher entropies are more likely to associate with IF. Biologically, such ‘chaos’ is possibly driven by the engagement of various components within the TME, namely the spatial intra-tumoral heterogeneity. We also note that while the H_SC_ scores in CT also appear to cluster in Cases 1A, 1B, 2, 4, and 5, the distribution in Case 3 is comparatively flatten and is similar to N; whereas the H_SC_ scores in N are normally flatten with lower magnitude, the distribution in Case 5 is intense and sharp. This phenomenon is possibly caused by the different infiltration level of lymphocytes. An efficient immune response can facilitate the recruitment of infiltrating T cells into the battlefield to either fuel the immunosuppression or promote immunoactivation, depending on the recruited T cell subtypes. Once the infiltration barriers (as seen in Figure 5C and Supplementary Figure S6) are broken, the immuno-architecture may tend to uniform and mitigate the spatial heterogeneity within TME.

#### Spatial Point Pattern Model Fitting

**FIGURE 7:**
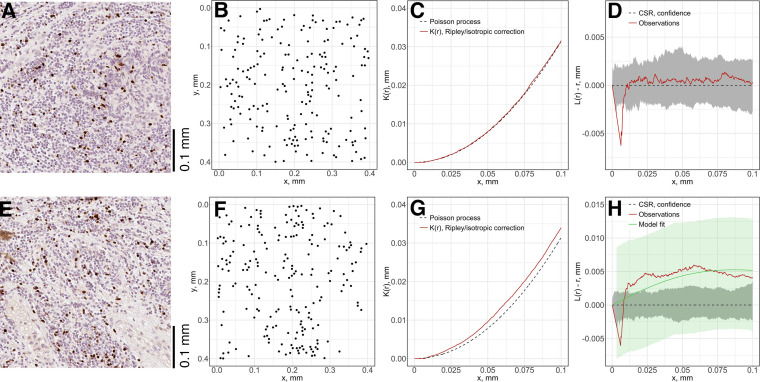
Local spatial point pattern analysis for subregions. We used a moving window to gauge local characteristics across each slide. **(A,E)** Original exemplar IHC subregions. **(B,F)** Associated point patterns obtained from image segmentation and coordinate extraction. **(C,G)** K-estimation using Ripley’s border correction for pattern **(B)** and **(F)**. Clark-Evans method is performed for CSR test, pattern (F) failed to pass the test and clustering model-fitting is performed. **(D,H)** L-transformation of *K* function and 95% confidence interval. For pattern **(B)**, the modified Thomas clustering process model is fit to pattern **(F)**. Results are evaluated using Dao-Genton goodness-of-fit test (green envelope).

For each subregion (Figures 7A,E), we perform the Complete Spatial Randomness (CSR) to check whether the associated point pattern (Figures 7B,F) follows homogeneous Poisson distribution (Figures 7C,G); if that window failed to pass the CSR test, we further fit the Thomas process model to the point pattern to quantify the clustering (Figures 7D,H). In this study, we either directly use these parameters, e.g., μ as the number of immune marker-labeled cells per cluster, or perform transformation for intuitive interpretation, e.g., σ^2^ is used to calculate the average distances of points to cluster areas and center.

As the window is moved through the slide, local features of spatial point patterns are quantified. Collective results are further classified based on their regions; therefore, the variance captures the intra-tumoral heterogeneity. Statistics for each region are also comparable among cases, which can reveal inter-tumoral heterogeneity. In this study, we use the following metrics to characterize each region for each case: mean cell density (counts/mm^2^), average number of cells per cluster, mean distance to cluster core, and mean cluster area. QCoD of the mean values for each parameter across cases are summarized in Table 1. They collectively reflect intra- and inter-tumoral heterogeneity.

**TABLE 1:** Quartile coefficient of dispersion (QCoD), Eq. 8, for spatial model fitting parameters (range and mean).

Marker	Region	Density	Cell/cluster	Mean distance	Cluster area
CD3	N	0.61–0.82 (0.67)	0.50–0.73 (0.64)	0.12–0.22 (0.16)	0.24–0.41 (0.31)
	IF	0.55–0.71 (0.64)	0.67–0.87 (0.77)	0.15–0.20 (0.17)	0.28–0.38 (0.32)
	CT	0.42–0.68 (0.57)	0.58–0.79 (0.71)	0.16–0.20 (0.18)	0.30–0.39 (0.34)
CD4	N	0.48–0.72 (0.62)	0.58–0.73 (0.64)	0.13–0.19 (0.17)	0.26–0.37 (0.32)
	IF	0.49–0.68 (0.57)	0.64–0.90 (0.76)	0.13–0.18 (0.15)	0.26–0.35 (0.30)
	CT	0.26–0.68 (0.53)	0.46–0.78 (0.65)	0.11–0.21 (0.16)	0.22–0.41 (0.32)
CD8	N	0.51–0.82 (0.61)	0.60–0.77 (0.67)	0.16–0.21 (0.18)	0.31–0.40 (0.35)
	IF	0.48–0.69 (0.59)	0.59–0.83 (0.71)	0.14–0.20 (0.17)	0.28–0.38 (0.33)
	CT	0.53–0.78 (0.64)	0.54–0.75 (0.66)	0.14–0.21 (0.18)	0.28–0.40 (0.35)
CD20	N	0.48–0.68 (0.59)	0.55–0.67 (0.60)	0.14–0.17 (0.15)	0.27–0.32 (0.29)
	IF	0.67–0.76 (0.71)	0.65–0.84 (0.78)	0.14–0.20 (0.17)	0.28–0.38 (0.33)
	CT	0.36–0.70 (0.50)	0.51–0.65 (0.61)	0.13–0.20 (0.16)	0.26–0.38 (0.32)
FoxP3	N	0.42–0.62 (0.48)	0.44–0.88 (0.60)	0.13–0.55 (0.23)	0.25–0.88 (0.42)
	IF	0.52–0.63 (0.58)	0.60–0.85 (0.73)	0.16–0.24 (0.19)	0.31–0.45 (0.36)
	CT	0.36–0.64 (0.50)	0.56–0.76 (0.67)	0.11–0.24 (0.18)	0.21–0.45 (0.35)

#### Cell Cluster Distributions and Morphometrics

We also extend our heterogeneity analysis to a global scale by quantifying cell clustering patterns. For each slide, the whole point pattern is clustered using a hierarchical clustering algorithm (Supplementary Figures S11A,B). For each detected cell cluster, we describe and characterize the shape by morphometrics, including convexity, circularity, and eccentricity. We first generate the alpha-hull (Supplementary Figure S11C) and convex hull from the point set that forms the cluster, upon which we derive the convexity and circularity; then we obtain the minor and major axes of the fitted ellipse (Supplementary Figure S11D) that covers 95% of the points of the cluster, from which we derive eccentricity.

**FIGURE 8:**
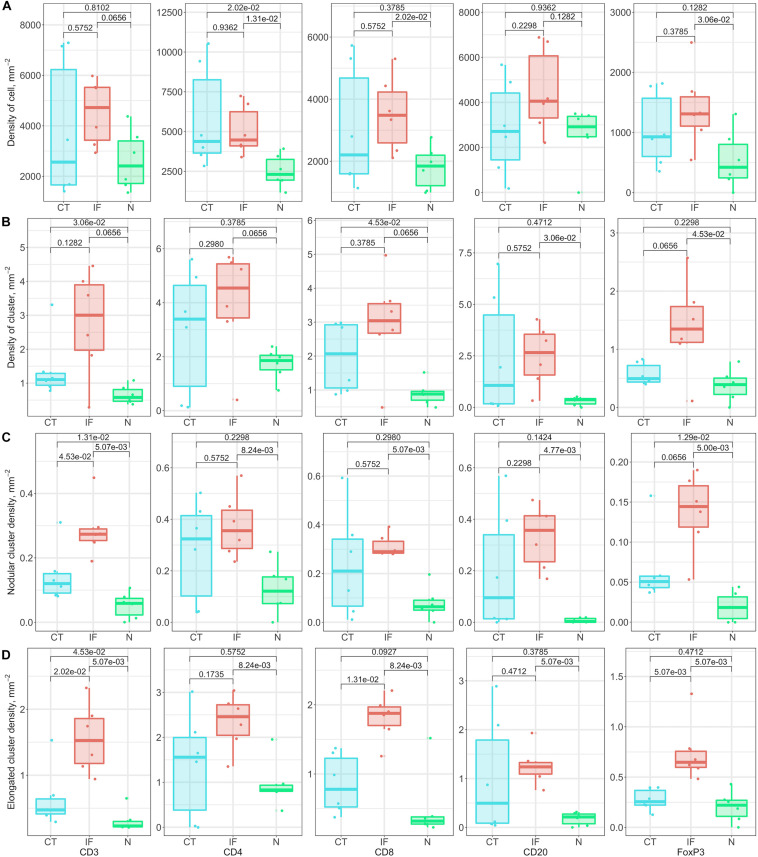
Relationship of cluster morphometrics between regions for specific markers. **(A)** Average density of corresponding immune cells within a cluster. **(B)** Density of clusters within specific regions. **(C)** Number of nodular clusters; standard: convexity > 0.8, circularity > 0.5, and eccentricity < 0.8. **(D)** Number of elongated clusters; standard: convexity < 0.3, or circularity < 0.3, or eccentricity > 0.9.

Similar to model-fitting analysis, the regional variations of morphometrics capture intra-tumoral heterogeneity, and variations among cases capture inter-tumoral heterogeneity. We use the following metrics to characterize regions, respectively: average density of cells within clusters, average density of clusters, nodular cluster density (Supplementary Figures S11E,G) and elongated cluster density (Supplementary Figures S11F,H). For each label, we use boxplots to show variations between cases, and we perform the Wilcoxon rank-sum test between groups. Figure 8 shows that even though IF is not always distinguished across labels and metrics, the nodular cluster densities and elongated cluster densities in IF are consistently higher than N. We also observe that CD3+ and CD8+ elongated cluster densities in IF are higher than CT. Such unique immuno-architecture may suggest that the elongated cytotoxic T cell cluster density in IF is a potential biomarker for further exploration.

### Correlation Analysis of the Spatial Patterns of Different Immune Markers

**FIGURE 9:**
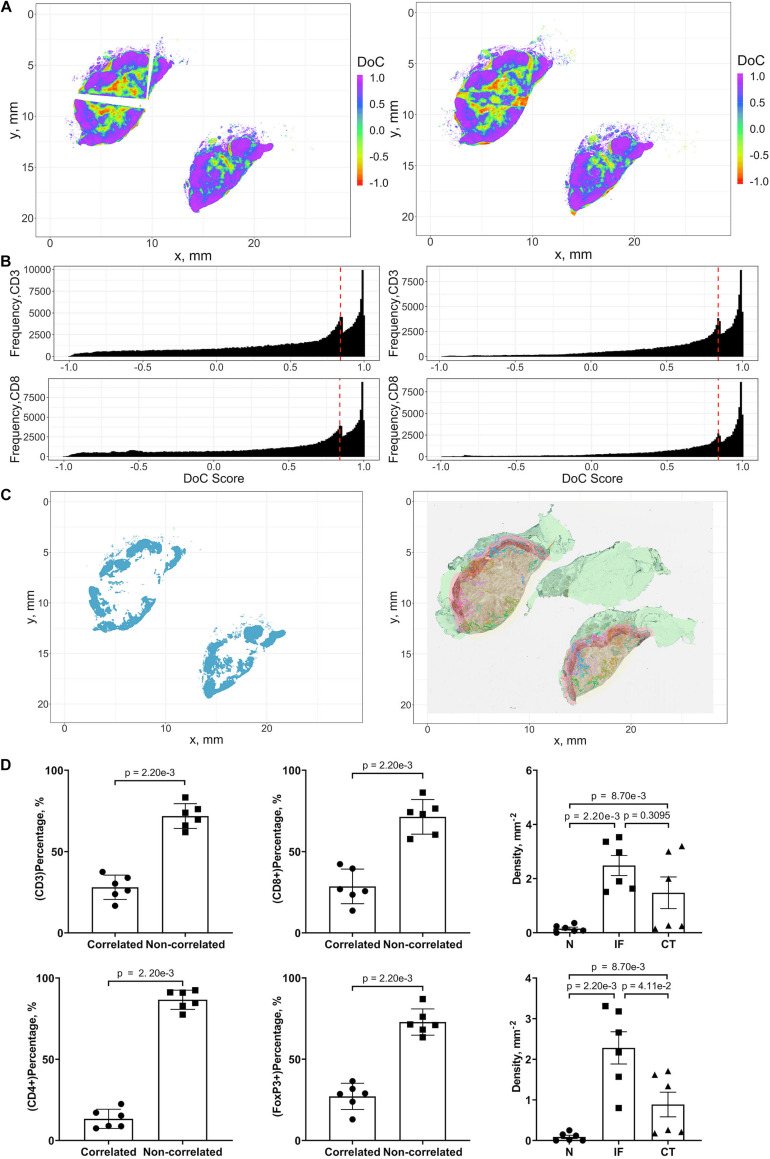
Results of positive correlation analysis. In this study, we analyze two pairs of markers: CD3+, CD8+ and CD4+, FoxP3+. Each marker within the correlation pair is defined as a “channel”. **(A)** DoC scores distributions for Cases 1A (left island) and 1B (right island). **(B)** Histograms of CD3+ and CD8+ DoC scores for Case 1A (left) and 1B (right). Red line is the predetermined threshold to select potential correlated cells. **(C)** Selected cells for both channels are mapped for clustering analysis (left) using HDBSCAN algorithm. We then define those clusters that both channels are involved as correlation hotspots (right, each cluster is represented by a colored outline). Green: normal tissue (N); Red: invasive front (IF); Yellow: central tumor (CT). **(D)** Statistical analysis. Top row: proportions of correlated CD3+ and CD8+ cells and density of correlated hotspots in different regions; bottom row: same metrics for CD4+ and FoxP3+.

The threshold for classification of the degree of colocalization (DoC) scores is determined by comparison of synthesized colocalization pairs. Considering the overall accuracy of our local registration algorithm is 0.976, we shift the point patterns of CD8+ left by 0.01/0.976 mm. This results in the majority (≥90%) of DoC for each individual point are larger than 0.84, hereby the threshold is established. In this study, two types of correlations are considered: positive correlation means co-occurrence of points of both channels are likely to be observed in subjects’ neighborhood. High DoC scores of points from both channels account for this type; negative correlation means co-occurrence of points of only one channel are likely to be observed in subject’s neighborhood. High DoC scores of one channel whereas low DoC scores of the other account for this type. Based on such criteria, we analyze the correlations between CD3+/CD8+, CD4+/FoxP3+, and CD8+/FoxP3+. Results are shown in Figure 9 with Cases 1A, B as an example, and clusters are represented by outlines in different colors.

#### Positive Correlations of CD3+ and CD8+ Immune Markers Identify Possible Anti-tumor Hotspots

For each case, the DoC scores, defined by Eq. 14, are assigned to each point of CD3+ and CD8+ markers. Next we use the threshold established above to select points with high DoC scores as candidates for clustering analysis using the same algorithm in cell clustering and morphometrics module (Figures 9A,B), and finally, the detected clusters that contain correlated cells from two channels are defined as hotspots of correlation and the cells within such hotspots are defined as correlated cells (Figure 9C).

We first examined the correlations between CD3+ and CD8+ marker pairs. As Table 2 shows, the numbers of correlated cells differ drastically from case to case, however, the ratios are relatively consistent across cases. The density of hotspots in the IF and CT are significantly higher than normal tissue (N) (Wilcoxon rank-sum ***p*** = **2.20e-3** and **8.70e-3**), but there is no difference between IF and CT (Wilcoxon rank-sum ***p*** = **0.3095**). Such distribution pattern of correlation clusters of CD3+ and CD8+ cells indicates possible sites of tumor infiltrate conferring anti-tumor immunity (Figure 9D).

**TABLE 2:** Statistical summary for CD3+ and CD8+ immune markers correlation analysis.

Case	QDoC (DoC Score)		Correlated cell counts		Percentage, %		Cluster density, mm^–^^2^		
	CD3	CD8	CD3	CD8	CD3	CD8	N	IF	CT
1A	0.95	0.94	71,630	66,097	23.9	25.7	0.36	3.36	2.00
1B	0.35	0.29	61,342	53,715	33.9	39.6	0.24	2.97	3.20
2	0.15	0.15	91,147	70,859	37.5	42.3	0.08	3.53	3.00
3	0.29	0.39	25,773	17,804	30.3	26.9	0	1.90	0.25
4	0.24	0.30	26,340	22,180	26.1	23.5	0.07	1.63	0.27
5	0.61	0.91	11,672	7,214	16.7	13.7	0.18	1.51	0.15

*N, normal tissue; IF, invasive front; CT, central tumor.*

#### Positive Correlations of CD4+ and FoxP3+ Immune Markers Identify Possible Immunosuppression Hotspots

The same workflow is repeated for the CD4+ and FoxP3+ pair. A summary of the results is presented in Table 3. Similar patterns are observed when compared to CD3+ and CD8+ statistics. However, the ratios of correlated cells are generally higher than CD3+ and CD8+ pairs. The density of hotspots within IF and CT are significantly higher than N (Wilcoxon rank-sum ***p*** = **2.20e-3** and **8.70e-3**), plus the density at IF is also significantly higher than CT (Wilcoxon rank-sum ***p*** = **4.11e-2**).

**TABLE 3:** Statistical summary for CD4+ and FoxP3+ immune markers correlation analysis.

Case	QDoC (DoC Score)		Correlated cell counts		Percentage, %		Cluster density, mm^–^^2^		
	CD4	FoxP3	CD4	FoxP3	CD4	FoxP3	N	IF	CT
1A	0.68	0.60	83,363	17,902	17.1	29.0	0.25	3.18	1.71
1B	0.40	0.36	65,267	14,719	22.5	36.5	0.12	3.31	1.62
2	0.52	0.53	59,281	15,630	15.3	23.9	0.02	2.17	1.34
3	1.30	0.39	25,172	12,085	9.0	31.8	0	2.66	0.26
4	0.85	0.90	19,385	6,547	8.8	13.0	0	0.80	0.21
5	0.87	0.49	18,596	7,586	7.5	28.6	0.15	1.57	0.18

*N, normal tissue; IF, invasive front; CT, central tumor.*

Collectively, our analysis identifies several hotspots in which CD3+/CD8+ and CD4+/FoxP3+ pairs are co-localized. The majority of identified hotspots are located within the IF. Such hotspots are characterized by strong correlations of CD3+/CD8+ and CD4/FoxP3+. These findings further reveal a strong heterogeneity within IF, as these two T cell subpopulations carry distinct immune characteristics.

#### Negative Correlations of CD8+ and FoxP3+ Immune Markers May Identify an Immune Response Landscape

We analyzed the negative correlation of CD8+ and FoxP3+ immune markers as a potential indicator of immune response landscape involving cytotoxic T cells (CTLs) and regulatory T cells (Tregs). In this section, two types of hotspots are defined. First, the hotspot with correlated CD8+ cells and non-correlated FoxP3+ cells; such clusters include FoxP3 cells that are surrounded by CD8+ cells, namely CD8-dominant hotspot. Biologically such hotspots may indicate places where CTLs may efficiently inhibit the strong immunosuppression of Tregs. Second is the opposite type, namely FoxP3-dominate hotspot, where the anti-tumor immunity is possibly impaired by Tregs.

The identification of such landscape can contribute to evaluating the immunotherapy treatment outcomes. Interactions are visualized by identifying negative correlation hotspots of two channels using previous workflow. Unlike the positive hotspots, negative hotspots are widespread throughout the entire tumor tissue for both types. For both types, the density of hotspots at CT and IF (Supplementary Table S3) are significantly higher than N (Wilcoxon rank-sum ***p*** = **1.52e-2** and **2.20e-3**) but no significant difference is observed between IF and CT (Wilcoxon rank-sum ***p*** = **0.0649**). For FoxP3-dominate hotspots, the CT and IF (Supplementary Table S4) are significantly higher than N (Wilcoxon rank-sum ***p*** = **1.52e-2** and **2.20e-3**) and the density in IF is also significantly higher than CT (Wilcoxon rank-sum ***p*** = **2.20e-3**). Results are shown in Supplementary Figure S12 with Cases 1 A, B as an example, and clusters are represented by outlines in different colors.

## Discussion

In this study, we proposed a digital pathology computational workflow to systematically analyze whole slide images (WSI) and quantify the intra- and inter-tumoral heterogeneity through multiple metrics. We analyzed immunohistochemistry (IHC) slides of tumor resections from five patients with TNBC. The sample size is limited to make inferences at the population level, but it is sufficient to look in-depth at each sample with multiple immune markers and to develop a methodology for spatial statistical characterization and build a platform that could be extended to large number of samples, including multiplex IHC and immunofluorescence microscopy (mIF). It should also be noted that each WSI contains enormous amount of information and large numbers of cells of different type to fulfill our need to obtain statistically and biologically meaningful results. In principle, this approach is consistent with personalized medicine where inferences could be made at the level of individual patient. For each patient, five biomarkers: CD3, CD4, CD8, CD20, and FoxP3, were labeled using IHC staining methods. The whole computational workflow starts with image processing: we use cell nucleus segmentation to obtain location information of labeled cells in their original slides and perform image registration using a multimodal protocol to calculate transformation matrices which map all slides to the reference CD4+ slide. We further proposed a pixel-distance based method, and with its application, we can characterize the whole slide into normal tissue (N), IF, and CT. From this point, all subsequent analysis results can be classified into tissue type/region categories to reveal intra-tumoral heterogeneity; for each region, we compare the results between cases for inter-tumoral heterogeneity.

By visualizing the density distributions for each slide and spatial entropy analysis, we identified significant spatial variations of cell densities within and across slides, which qualitatively characterized the intra- and inter-tumoral heterogeneity. In addition, we are particularly interested in the spatial profiles along the direction from N through the IF to the innermost of CT. We observe that for each slide, the cell densities increase sharply within IF and then drop, in some cases to a plateau and in some precipitously and exhibiting fluctuations; hence corroborating the distinct role of IF in the immuno-architecture. This allows us to propose a hypothesis that the source of the immune cells in the IF is not in the normal tissue into which the tumor grows, but rather the cells extravasate from the tumor vasculature whose density is known to be higher at the rim of the tumor (Stamatelos et al., 2019); this hypothesis needs to be tested in future studies. We then fit a spatial point process model to data within subregions to capture local variabilities. Statistical results indicate that strong intra- and inter-tumoral heterogeneities co-exist across our study cohort. For each slide, we also evaluated the cell clustering using a hierarchical based algorithm against full point patterns. We then gauged the first-order properties and morphometrics of each cluster. Results revealed that variations are more likely to occur in CT and IF but less likely in N. We also identified a unique distribution pattern of nodular cytotoxic T cell clusters. As our recent study has shown (Gong et al., 2018), the distribution and shape of clusters have certain relations to the treatment outcomes, thus our findings may lead to predictive biomarkers that could eventually be used clinically when tested on a large number of specimens. Finally, we performed correlation analysis and discovered that the IF is multifaceted and may bear pro- and anti-tumor functions simultaneously, e.g., with higher expressions of CD8+ and FoxP3+ cells.

In addition to characterizing intra- and inter-tumoral heterogeneity, the characteristics obtained from tissue samples, such as spatial cell density profiles of different immune cells, the magnitude of spatial cell density fluctuations, and the spatial correlations between the densities of different immune cell types, can facilitate development and parameterization of spatially resolved computational immuno-oncology models. Recently, QSP models have been applied to immuno-oncology research as a platform for conducting virtual clinical trials (Cheng et al., 2017; Bai et al., 2019; Jafarnejad et al., 2019; Milberg et al., 2019; Ma et al., 2020). These models capture system scale behavior in cancer patients and are capable of population level predictions of disease trajectories in response to intervention. On tissue-cellular scale, ABMs have been employed and used for spatially explicit simulations to investigate emergent behavior arising from interactions between cancer and immune cells, such as spatial and spatio-temporal variations in tumor morphology and immuno-architecture (Kim et al., 2009; Shi et al., 2014; Wells et al., 2015; Gong et al., 2017; Norton et al., 2017, 2019; Pourhasanzade et al., 2017; Hoehme et al., 2018; Ji et al., 2019). When combining QSP models with ABM, cancer models can be further enhanced by taking advantage of both model types: while the QSP module captures whole-body temporal dynamics including lymph nodes, blood, peripheral compartment, and tumor, ABM simulation accounts for crucial aspects of high-granularity features such as cancer cell clonal evolution and TME heterogeneity. The resulting hybrid model will be able to closely track and predict the course of cancer development, both primary tumors and metastases, and potentially during treatment in individual patients by incorporating patient-specific TME characteristics, which can be quantified using our digital pathology platform. Such synergy would enable a better understanding of impact of spatial heterogeneities in the CT and IF on the pathophysiological parameters and variables. Strictly speaking, cell densities calculated directly from digitally segmented pathology images as described in this study represent the number of cell profiles per unit area in the tissue slide typically with a 4–5 micron thickness, which is a common metric in pathology, where a cell signature is a section of cell with an area larger than the detectable threshold set in our segmentation algorithm. However, in computational models the cell concentrations are usually represented as the number of cells per unit volume rather than unit area. Using methods from the field of stereology (Weibel et al., 1966), 3D numerical densities (*N*_V_) can be estimated from 2D density (*N*_A_) using the following equation: *N*_V_ = *N*_A_/(*t* + *D* − 2*h*), where *t* is the thickness of the section, *D* is the diameter of stained cells (which are lymphocytes in the scope of this study), and *h* is the minimum height of detectable spherical cap (which can be derived from cellular segmentation algorithm parameters) (Royet, 1991). In this equation, 2*h* in the denominator accounts for loss of undetected parts of the cell. *N*_V_ indicates number of cells per unit volume and can directly be used to inform 3D spatial models of tumor-immune interactions. Using this equation, one could convert the 2D densities (in mm^–2^) to 3D densities (in mm^–3^); in the conversion the slide thickness is typically *t* = 4.5–5 μm, *h* = $D/2-\sqrt{{\left(D/2\right)}^{2}-A_{c\,r\,i\,t}/\pi }$, where *A*_*crit*_ is the minimum area detectable during the segmentation, typically ∼10 μm^2^; diameter values reported for T cell (5–7.1 μm) and for B cell (5.5–9 μm) are also necessary for the conversion (Chapman et al., 1981; Turgeon, 2005; Tsourkas et al., 2007; Strokotov et al., 2009; El Hentati et al., 2010; Mrozek-Gorska et al., 2019; Renner et al., 2020).

Depending on the purpose of each computer simulation, one can either derive overall 3D density and use it to populate the *in silico* TME; or if spatial heterogeneity is of interest, the variability of cell density can be taken into account by sampling multiple *N*_A_ from different regions of the digital pathology analysis output to initiate the simulated TME with a range of *N*_V_ values in space. After simulation, the same methods employed in this study to analyze spatial correlations between different cell types can be applied to virtual sections of model-generated three-dimensional tumor, which would enable quantitative comparisons between model-generated spatial patterns of cancer and immune cells and patient pathology images. QSP and ABM have been used to model the tumor growth and invasion of several cancer types, such as melanoma (Wang et al., 2013; Milberg et al., 2019), breast cancer (Bates et al., 2006; Bianca and Pennisi, 2012), colorectal (Kather et al., 2017), and non-small cell lung cancer (Jafarnejad et al., 2019).

Future work should focus on increasing the scale of the current workflow. In this study, cells expressing CD8/FoxP3 are considered as cytotoxic/regulatory T cells. Such loose criterion serves the need to test the functionality of the workflow using preliminary computational results from pathology images. However, a comprehensive biomarker panel is required to account for the complexity in cell lineage definition and further to characterize the components in TME. Such materials will be obtained in subsequent studies applied to multiplex labeled specimens. Improving the performance of image processing is another critical issue. We recognize the power of artificial intelligence in digital pathology and such techniques could be incorporated in an extension of the workflow. For example, traditional segmentation algorithms may not adequately distinguish cell boundaries due to staining issues. A possibility is to introduce convolutional neural network (CNN) trained on well-defined ground-truth (Khosravi et al., 2018). In this study, immune markers are stained on consecutive slices of tumor resections, therefore artifacts may be introduced such as distortions. Registration reduces uncertainty introduced by these artifacts, but cannot fully compensate for the location mismatch, and errors may be introduced in derived point patterns and subsequent analysis. Such problems can be alleviated by harvesting data from multiplex images in the first place by labeling different cells and molecules on the same slide; in this case artificial location shifts, sample folds and z-axis differences are essentially eliminated. In the tissue type characterization step, we identified the IF by averaging annotations provided by expert pathologist. To pinpoint IF, deep learning methods can be applied for automated tissue segmentation. In point pattern analysis stage, we extract copious intra- and inter-tumoral heterogeneity information from collective slides; when correlated with treatment outcomes, these results can provide more useful information for pathologists and immuno-oncologists.

## Data Availability Statement

The datasets generated or analyzed during this work are available from the corresponding author on reasonable request. The codes for computational methods are made available at https://github.com/popellab/SpatHeterogeneity-TNBC. The open source software QuPath may be downloaded at https://qupath.github.io/. The open source software Blender may be downloaded at https://www.blender.org/download/. The open source software Icy may be downloaded at http://icy.bioimageanalysis.org/download/.

## Ethics Statement

The studies involving human participants were reviewed and approved by Institutional Review Board of the Johns Hopkins Medical Institutions. Written informed consent for participation was not required for this study in accordance with the national legislation and the institutional requirements.

## Author Contributions

HM, CG, and AP designed the workflow. HM processed the images, implemented the statistical tests, and produced the results. AC-M provided the specimens, assisted in the interpretation of results, and edited the manuscript. HM, CG, JS, EF, AS, EJ, VS, LE, AC-M, and AP critically edited the manuscript. All authors contributed to the article and approved the submitted version.

## Conflict of Interest

The authors declare that the research was conducted in the absence of any commercial or financial relationships that could be construed as a potential conflict of interest.
