# Supplementary material for: Digital Pathology Analysis Quantifies Spatial Heterogeneity of CD3, CD4, CD8, CD20, and FoxP3 Immune Markers in Triple-Negative Breast Cancer
Source: Front Physiol. 2020 Oct 19;11:583333. doi: 10.3389/fphys.2020.583333 (PMC7604437; doi:10.3389/fphys.2020.583333)
Supplement: Supplementary file 1 [file Data_Sheet_1.docx]

Supplementary Material

**
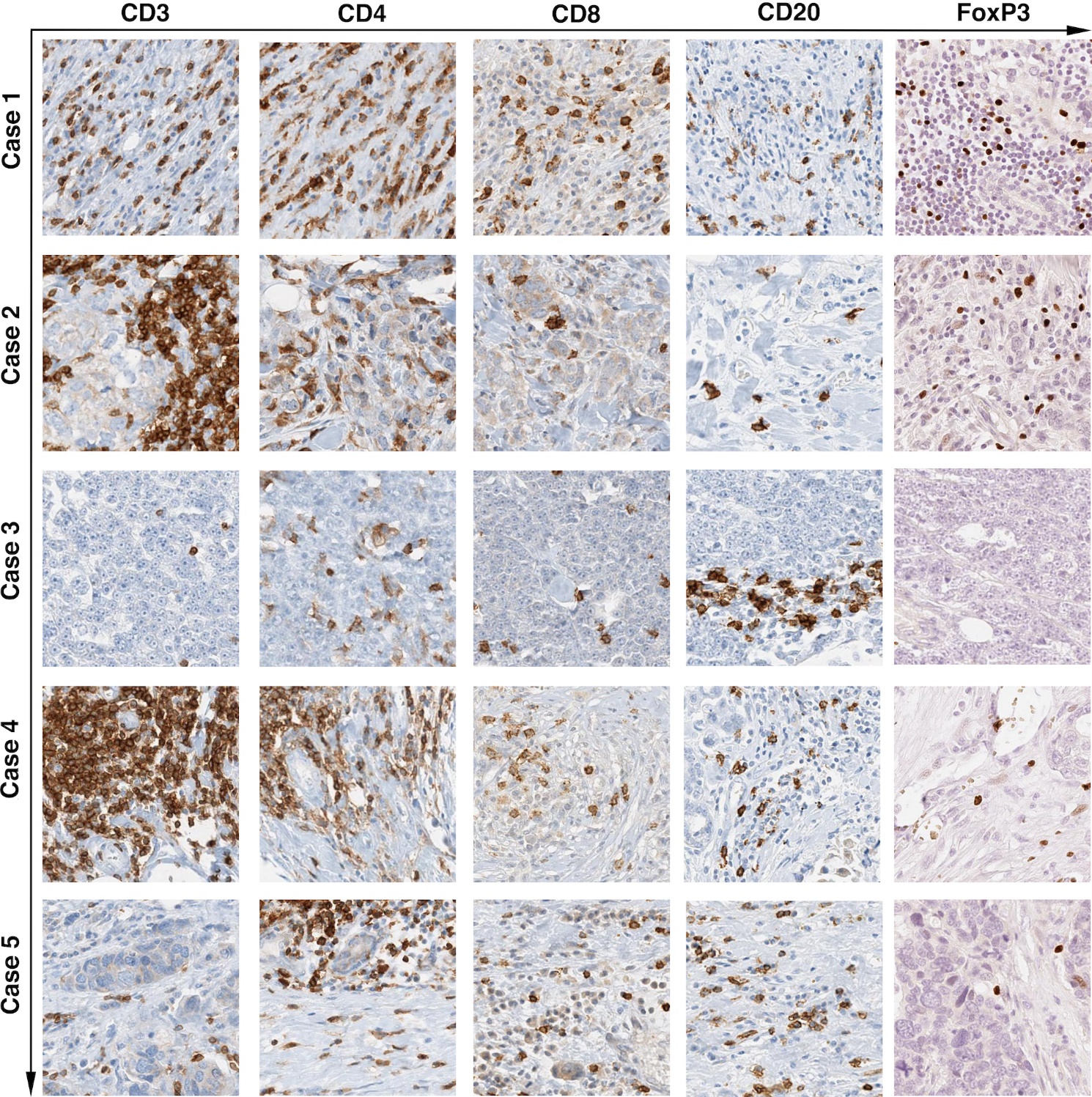
**

**Figure S1.** **Representative subregions (tiles) panel for all cases.** Tiles are segmented using custom Python algorithm and then fed to the customized Matlab algorithm for cell detection. The size of each tile is 200µm × 200µm (400 pixels × 400 pixels).


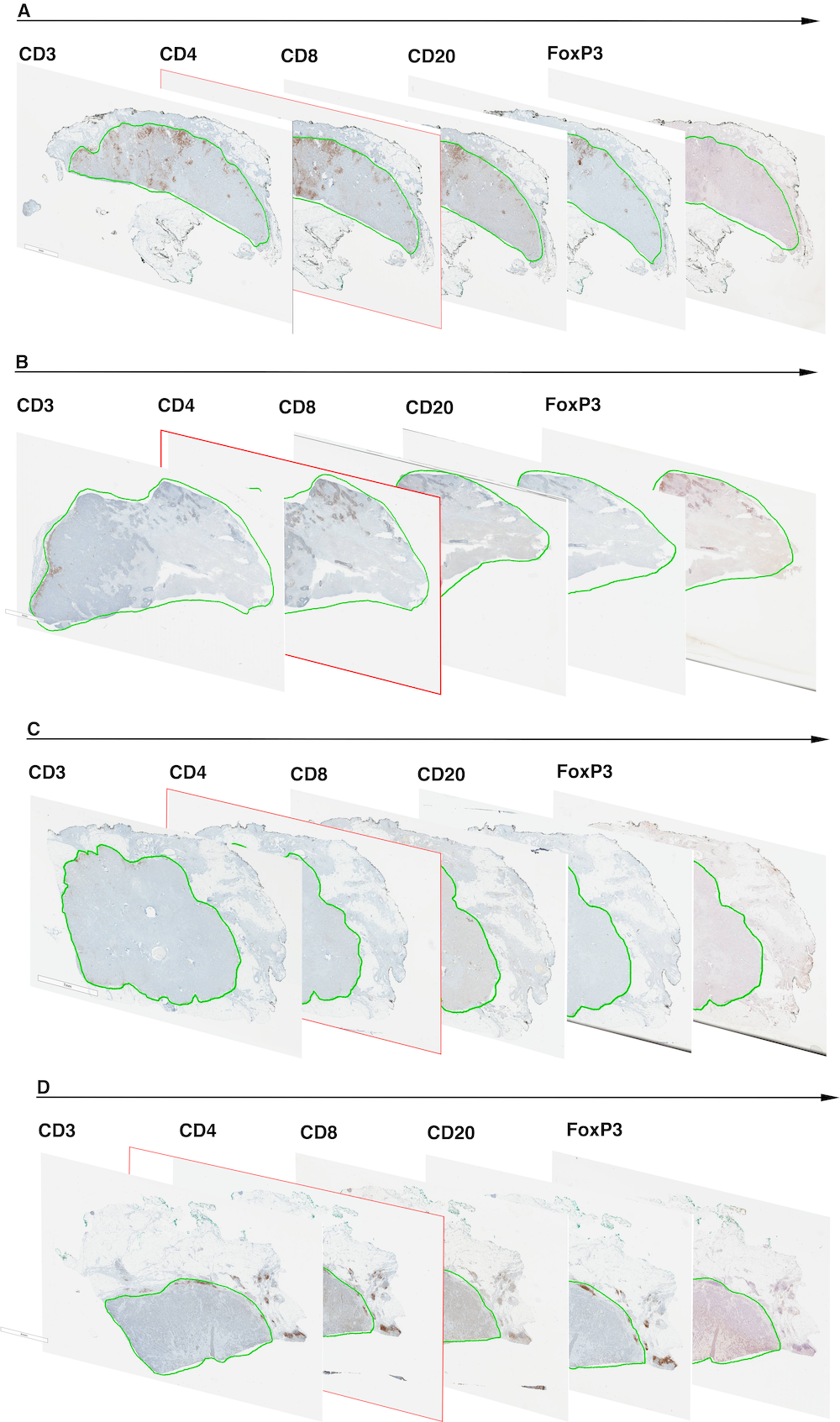


**Figure S2.** Whole slide tissue panels with breast cancer pathologist annotated outline (annotations, indicated by green contours) for **(A)** Case 2; **(B)** Case 3; **(C)** Case 4; and **(D)** Case 5. Red border indicates the reference slide for image registration.

**IHC image segmentation using QuPath**

We use open-source digital pathology analysis software QuPath to perform the nucleus segmentation of IHC slides. The workflow has three steps: preprocessing, nucleus detection, and cell classification and is carried out for each IHC staining set. To start, whole slide images (WSIs) are imported into QuPath. To compensate for the staining variance across the slides, we first find a representative region containing well-stained tissue as well as background and then draw a rectangle annotation by clicking the *Rectangle* button under *Tools* button in the toolbar. Then, we run the *Analyze → Preprocessing → Estimate stain vectors* command and then select *yes* if QuPath suggests updating the background values. This operation prompts a pop-up window to visualize the stain vector. We click the *Auto* button to automatically adjust and then the updated stain vector is saved for future use. In the second step, we first run the *Classify → Training images → create region annotations* command to randomly choose 75 subregions across Case 1, 2, and 3 slides (25 subregions each) to construct a training set. All subregions are combined into an integrated image by running *Classify → Training images → create combined training image* command. Next, all nucleus in the combined image, regardless of cell types, are detected by running the *Analyze → Cell detection → cell detection* command. The boundaries of nucleus are then expanded outwards for 7.5 µm to construct pseudo cell objects. QuPath achieves cell classification based on a list of intensity and morphometry measurements, which are calculated from the previous step. To facilitate this process, we smooth the existing measurements with the *Analyze → Calculate features→ Add smoothed features* command by weighted averaging of the corresponding measurements of neighboring cells. In the third step, significant amounts of cells are manually annotated into three categories: ‘immune cells’, ‘others’ (non-stained cells) and ignore (artifacts) with the polygons options under *Tools* menu to improve the accuracy of the automatic classification. This process is interactive by running the *Classify → Object classifications → train object classifier* command, and the classification result is updated once an annotation is drawn. Subsequently, the remaining IHC slides (Cases 4 and 5) will serve as the validation set to test the performance of the classifier, and the classification results are evaluated by eye. While this preliminary assessment ensures the classifier is operational, quantitative validation should also be performed to confirm the validity.

**Algorithm performance evaluation**

To further validate the performance of QuPath on our samples, 20 subregions from one slide for each case are sampled using the system sampling method, labeled cells are manually detected, and then the sensitivity and precision of our algorithm are evaluated. For each subregion, the number of cells that are detected both by algorithm and manual approach is denoted $n_{i}$ (true positive, TP), where $i$ refers to the case number; the number of cells that are detected by algorithm but rejected in manual counting is denoted $p_{i}$ (false positive, FP); the number of cells that are detected manually but left out by the algorithm is denoted $q_{i}$(false negative, FN). Now the sensitivity (S) and precision (P) can be calculated as:

$\boldsymbol{S =}\frac{\boldsymbol{TP}}{\boldsymbol{TP + FN}}\boldsymbol{=}\frac{\boldsymbol{\Sigma}_{\boldsymbol{i}}\boldsymbol{n}_{\boldsymbol{i}}}{\boldsymbol{\Sigma}_{\boldsymbol{i}}\boldsymbol{n}_{\boldsymbol{i}}\boldsymbol{+}\boldsymbol{\Sigma}_{\boldsymbol{i}}\boldsymbol{q}_{\boldsymbol{i}}}$ **(S2)**

$\boldsymbol{P =}\frac{\boldsymbol{TP}}{\boldsymbol{TP + FP}}\boldsymbol{=}\frac{\boldsymbol{\Sigma}_{\boldsymbol{i}}\boldsymbol{n}_{\boldsymbol{i}}}{\boldsymbol{\Sigma}_{\boldsymbol{i}}\boldsymbol{n}_{\boldsymbol{i}}\boldsymbol{+}\boldsymbol{\Sigma}_{\boldsymbol{i}}\boldsymbol{p}_{\boldsymbol{i}}}$ **(S3)**

The standard error of the mean associated with each metric for overall data can be calculated:

$\boldsymbol{s}\boldsymbol{e}_{\boldsymbol{S}}\boldsymbol{=}\sqrt{\frac{\boldsymbol{S(1-S)}}{\boldsymbol{TP + FN}}}$ **(S4)**

$\boldsymbol{s}\boldsymbol{e}_{\boldsymbol{P}}\boldsymbol{=}\sqrt{\frac{\boldsymbol{P(1-P)}}{\boldsymbol{TP + FP}}}$ **(S5)**

According to aforementioned metrics, we identified *TP* = 10,573; *FP* = 2,270; *FN* = 822. Using equations (S2) - (S5), we computed the precision (P) and sensitivity (S) indexes of the algorithm to be *S* = 92.8 ± 0.2% and *P* = 82.3 ± 0.3. In practice, the QuPath’s segmentation algorithm is tuned to be sensitive to the color variance in yellow channel, which reduces the under-estimation rates in cell-dense regions (represented by low FN). Meanwhile, high sensitivity to yellow channel leads to the over-estimation in non-dense regions as the algorithm by false classification of non-cell objects as they hold similar patterns in color space (represented by FP). Spearman’s rank correlation coefficient ($\rho$) is computed to test the correlation level between algorithm and manual approach, the calculation is performed using function ‘cor.test’ with argument ‘spearman’ in R package ‘stats’ (Team, 2015). $\rho$ is calculated to be 0.978, suggesting a relatively strong agreement between manual approach and the algorithm. Full parameter values and feature lists to set up the QuPath algorithms are presented in Table S1 - 2 and summarized evaluation statistics are listed in Table S5.

In conclusion, we have successfully trained and validated 5 classifiers for 5 IHC biomarkers (CD3, CD4, CD8, CD20, and FoxP3) using QuPath and the pipeline can be readily replicated. The numbers of detected cells for each label ranges from 12,857 (Case 3, CD20+) to 424,807 (Case 1A, CD4+). Fig. S2 shows the detection results for four subregions. Detailed immune parameters detected by the algorithm for each specimen are listed in Table S6. Such data are also compared with previously published findings on all specimens from the cohort (Cimino-Mathews et al., 2016) and statistical agreements are observed, which further corroborate the validity of QuPath on our slides.

**Image registration methods**

Before registration, reference and target slides are downsampled to blur the texture details. Then the downsampled moving image (to be registered) is first segmented into multiple subregions and the fixed (reference) image is also segmented to include the same area. Next, the speeded-up robust feature (SURF) registration algorithm from Matlab is used to match the features detected from both images for alignment (Qidwai and Chen, 2009).

Automatic registration applies to most cases but not all. Image downsampling reduces the texture details, but also the contour details. Additionally, contour features are not consistent since the slides are obtained from different z-slices. To improve registration performance, we applied a manual registration algorithm from the open-source platform Icy (De Chaumont et al., 2012). Human-eye detected feature pairs are manually selected, and the one on the fixed image is labeled to generate projection on the moving image. The projection should be relocated to the feature position for the rigid registration. The performance of registration is determined by the quality and quantity of feature pairs (how approximate the two features represent the same location). In this study, 5 pairs are subjects to global registration and 15 pairs are subjects to local registration.

**Registration robustness test**

To test the robustness of the registration algorithm, we measure the performance of local image registration versus global registration. Benchmarking of traditional evaluation methods (Borovec et al., 2018) suggests using the differences of image intensities between the reference and the transformed source to represent the registration accuracy. However, as a recent study pointed out (Wang et al., 2014), these criteria can be misleading when applied to medical images, since the intensity value of the pixel in the reference image and the one of wrongly registered pixel may be similar due to similarity of textures at local level and the intensity value of the pixel in the reference image and the one of correctly registered pixel may be distinct due to markers stained with different colors. Therefore, we used Dice similarity coefficient (DSC) in this study to gauge the agreement level of shapes between reference tissues and target tissues. The underlying principle is straightforward: given the contours of reference and target images, the DSC is determined as:

$\boldsymbol{DSC =}\frac{\boldsymbol{2\times|R\cap T|}}{\boldsymbol{|R| + |T|}}$ **(S6)**

where |*R*| is the area of reference contour; |*T*| is the area of target contour; |*R|*+|*T*| is the area of overlapping regions.

According to the definition above, when reference and target images are perfectly matched, the area of intersections should equal to two times the sum, thus the DSC score is 1; when reference and target images are poorly matched, then two contours seldomly intersect, which leads to the DSC score approaching 0. Therefore, the DSC score is defined within [0, 1] interval and higher values indicate better registration accuracy.

Results show that the performance of local registration (DSC mean = 0.917) is significantly better than global registration (DSC mean = 0.876), however, the global registration of those 5 pairs (DSC mean = 0.916) reveals results comparable to local registration.

**Fractal analysis**

In order to best determine the intra-tumoral heterogeneity using the spatial statistics methods, we assessed the effect of window size on spatial model fitting parameters and the number of clustered point patterns. Five different labels originated from five different cases are selected to ensure robustness: Case 1A: CD3+; Case 1B: CD3+; Case 2: CD4+; Case 3: CD8+; Case 4: CD20+; Case 5: FoxP3+.

The point pattern associated with each label was selected for heterogeneity analysis: window sizes ranging from 0.1 to 5mm (with a step size equals half of the window size) are used for spatial point process model fitting. All cases reveal the same pattern: the number of clusters reaches maximum in the range 0.3 – 0.5 mm and then exhibits gradual decrease. Given the large density of labeled cells for all labels, underestimation of density variation is avoided for small window size. Subsequent decrease of cluster numbers is due to the increase of window size so that the number of windows available for analysis is reduced. For each chosen window size, the quartile coefficient of dispersion (QCoD) is calculated. As the window size increases, QCoD experiences drastic fluctuations once the window size passes 1 $mm$, but for most cases it is relatively stable at smaller window sizes. For Cases 1A, 1B, 2, and 4, the QCoD even reach the global minimum. The acceptable window size should be balanced so that local density variation is properly considered, and simultaneously global variability among different windows is maintained. Based on these considerations, we select 0.4mm as the window size for spatial point pattern analysis.


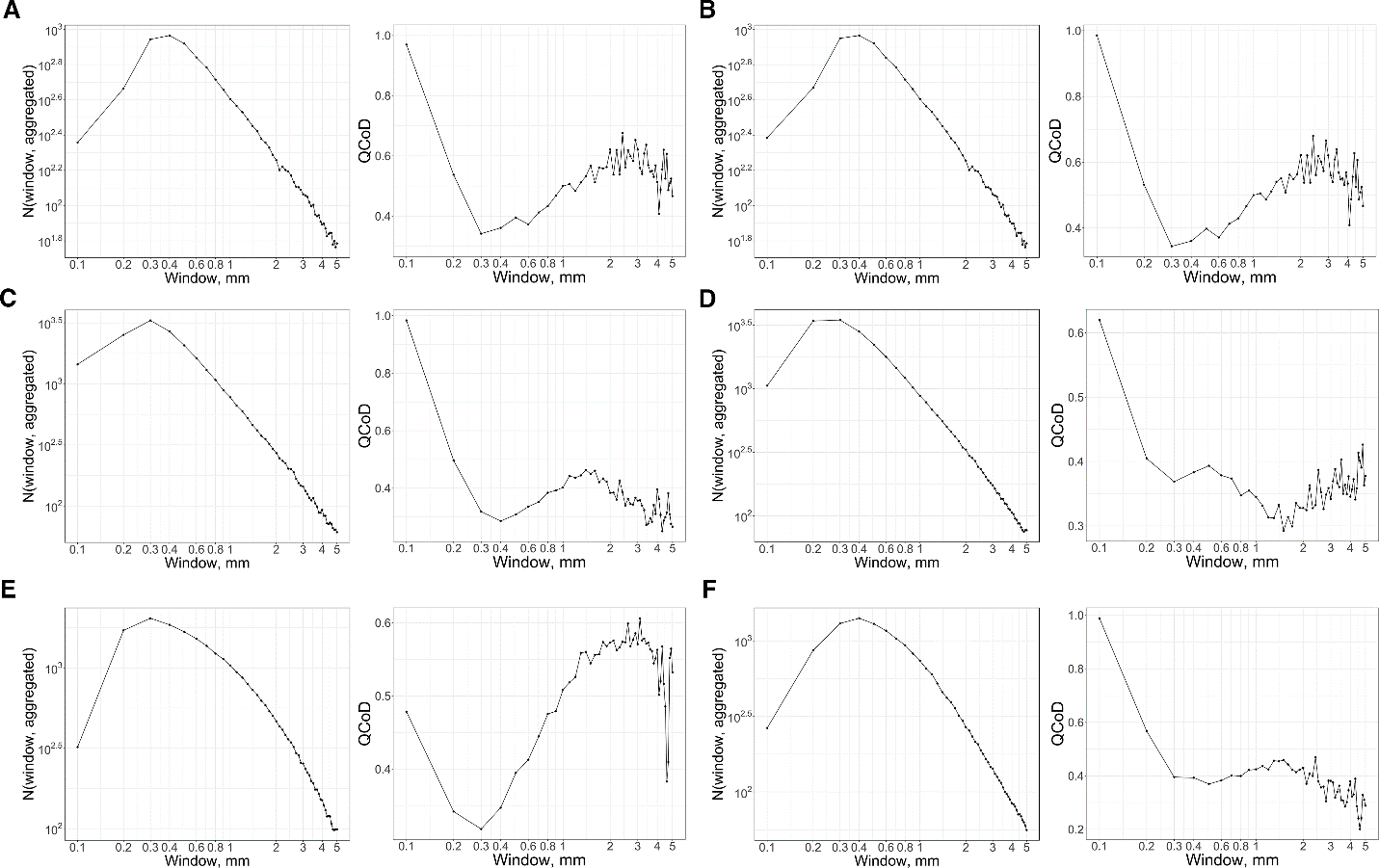


**Figure S3. The effect of window size on the total number of clustered subregions and global variability.** For each case, we select one label as subject for analysis. **(A)** Case 1A; **(B)** Case 1B; **(C)** Case 2; **(D)** Case 3; **(E)** Case 4; **(F)** Case 5. For each case, right panel is the Quartile coefficient of dispersion (QCoD) of fitted cluster radii and left panel is the number of fitted clusters, at the corresponding window size.

**
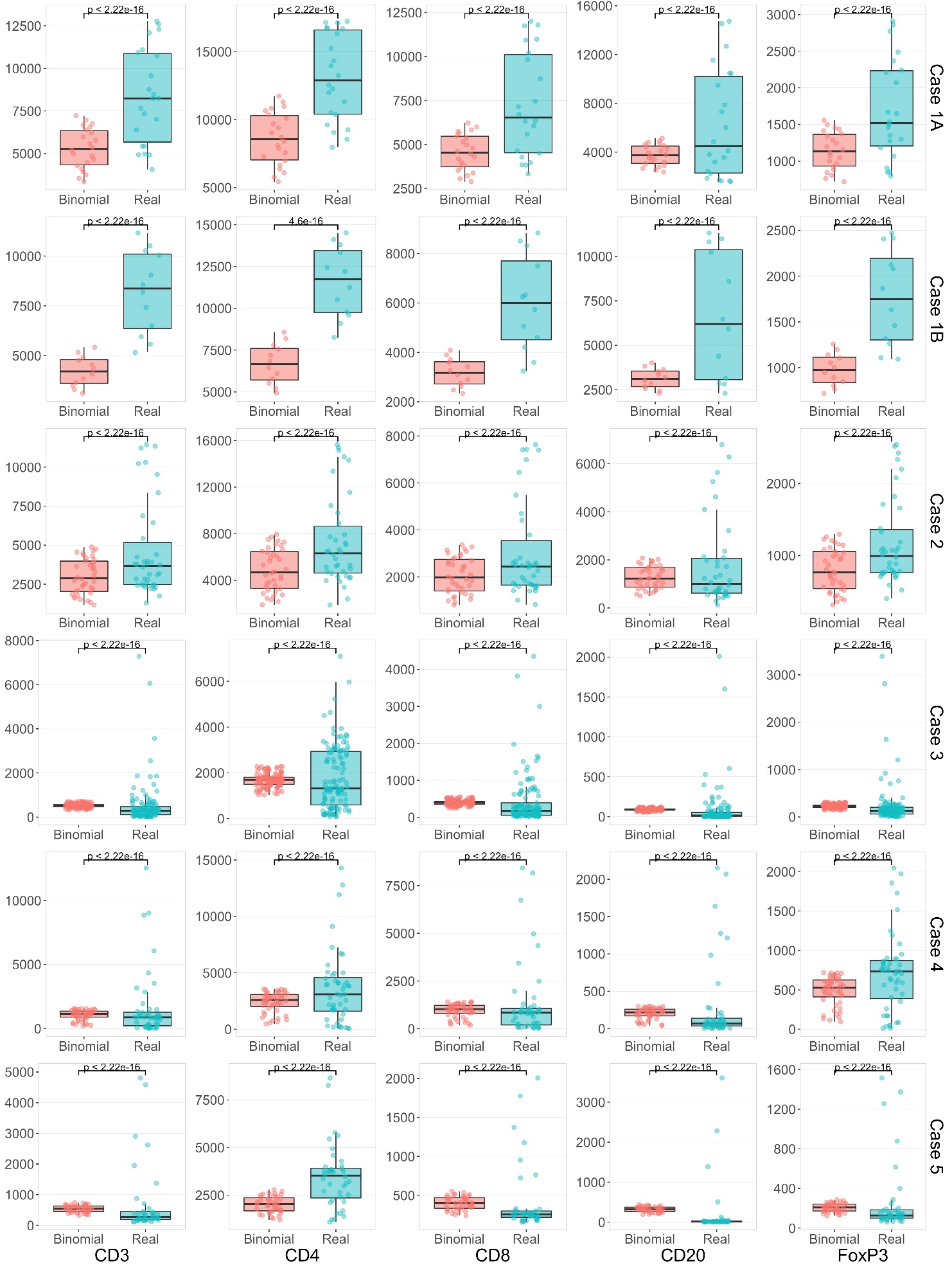
**

**Figure S4. Distribution patterns of cell counts across distance-based sections across whole tissue and chi-square test results.** To corroborate that the observed infiltration profiles are driven by the tumor spatial heterogeneity, we compare the actual cell counts (frequencies) to expected number in each section. We assume the number of cells in each section follows binomial distribution so that for each section, the expected number, n, can be calculated as: n = N×P, where N is the total number of cells, and P is the probability that a point falls within the section. P can be derived from the area of section/total area. Results show that all trials reject the null hypothesis (**p** < **1e-3**), therefore confirm that the infiltration pattern is a realization of tumor heterogeneity rather than random process.

**
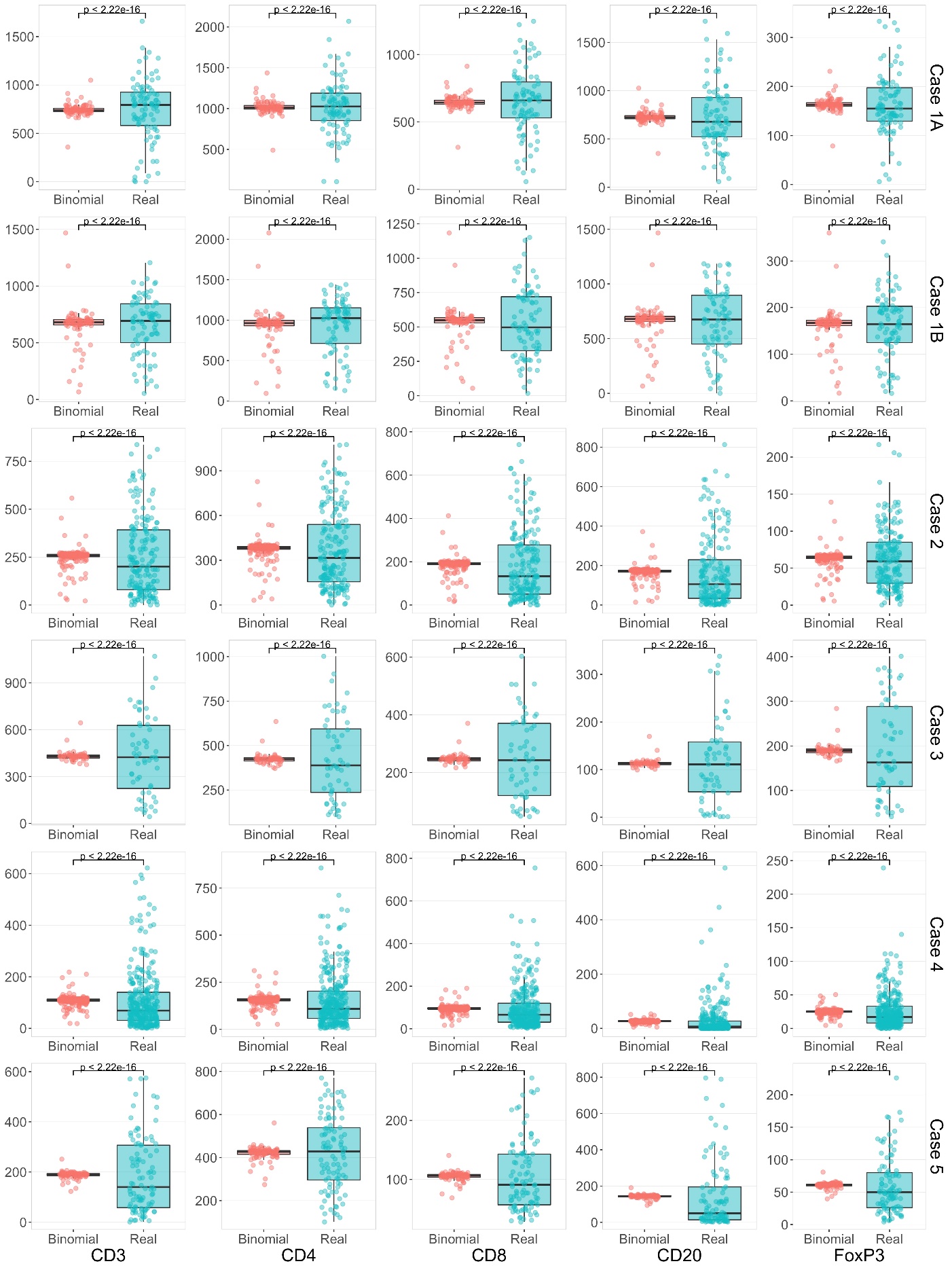
**

**Figure S5. Distribution patterns of cell counts across distance-based sections in invasive front and chi-square test results.** Results also show that all trials reject the null hypothesis (**p** < **1e-3**), therefore confirm that the infiltration pattern in invasive front is also a realization of tumor heterogeneity.

**
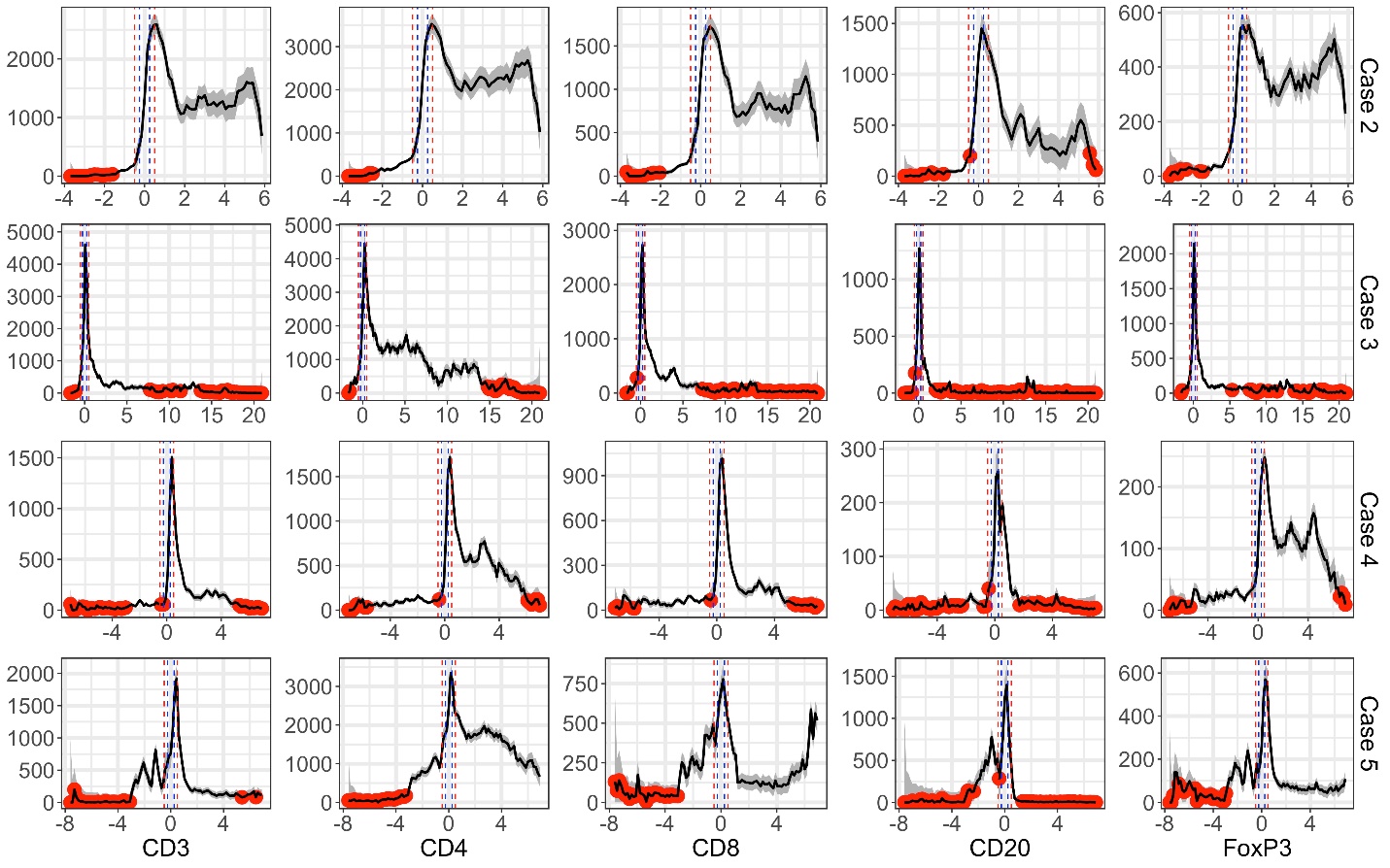
**

**Figure S6. Cell density-distance profile for Cases 2-5 with 95% CI.** Blue and red lines indicate 0.5 and 1mm wide invasive front. Red dots label locations where density value is not reliable.

**
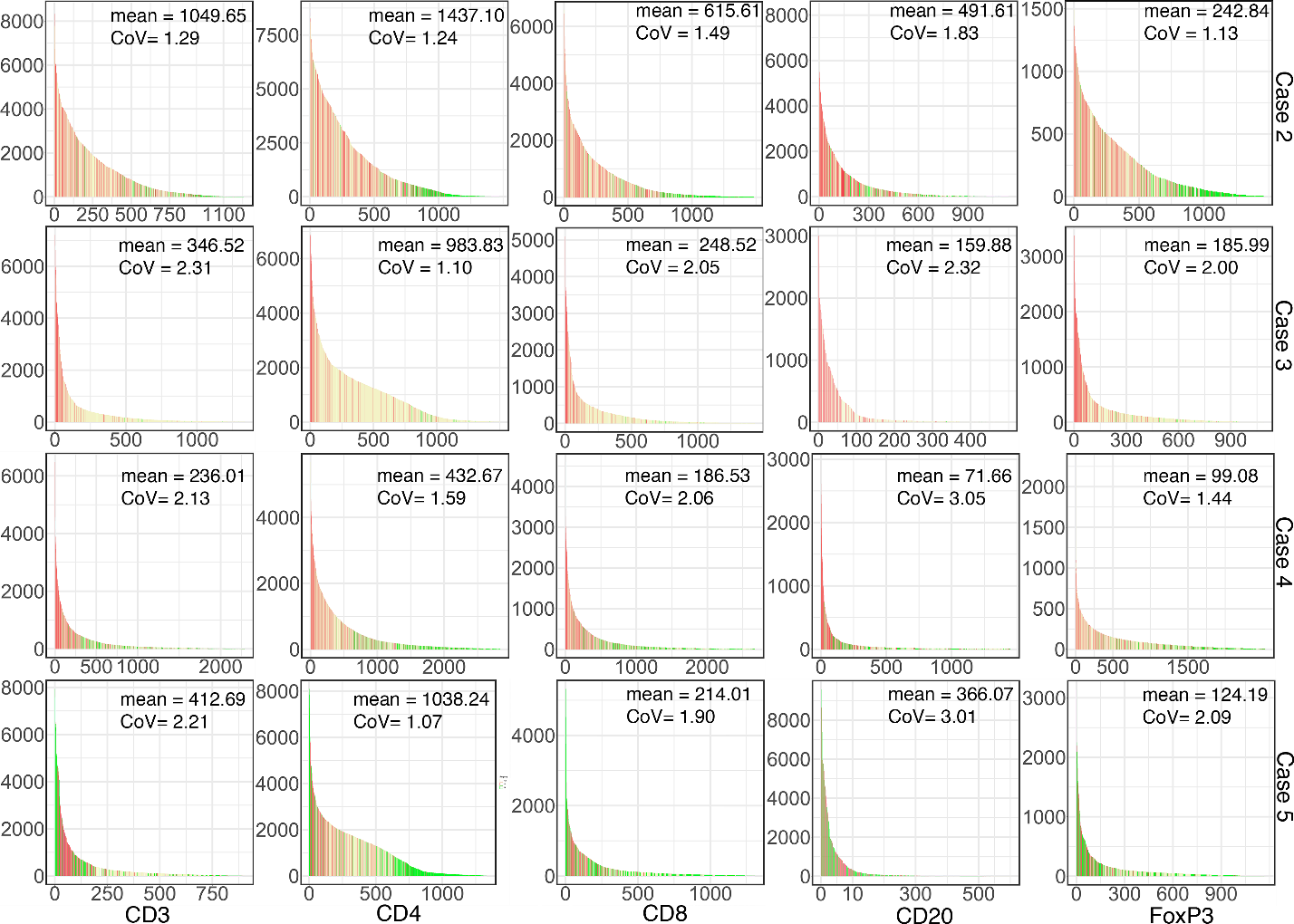
**

**Figure S7. Waterfall plots for cell densities in subregions in Cases 2-5 with statistics.** Green: normal tissue; red: invasive front; yellow: cancer tissue.


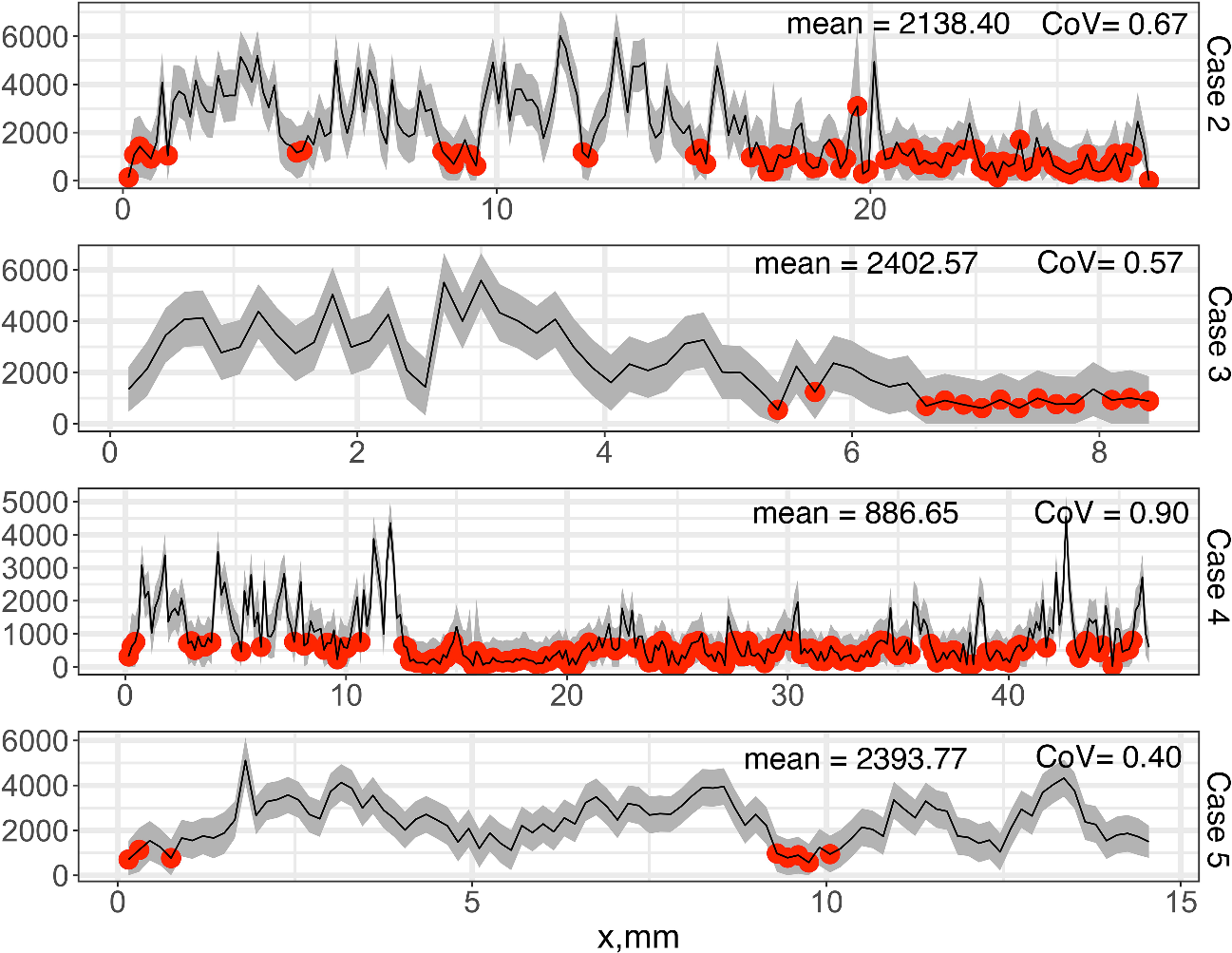


**Figure S8.** **Cell density-distance profiles for Cases 2-5 of CD4+ in invasive front with 95% CI.** Red dots label locations where density value is not reliable.

**
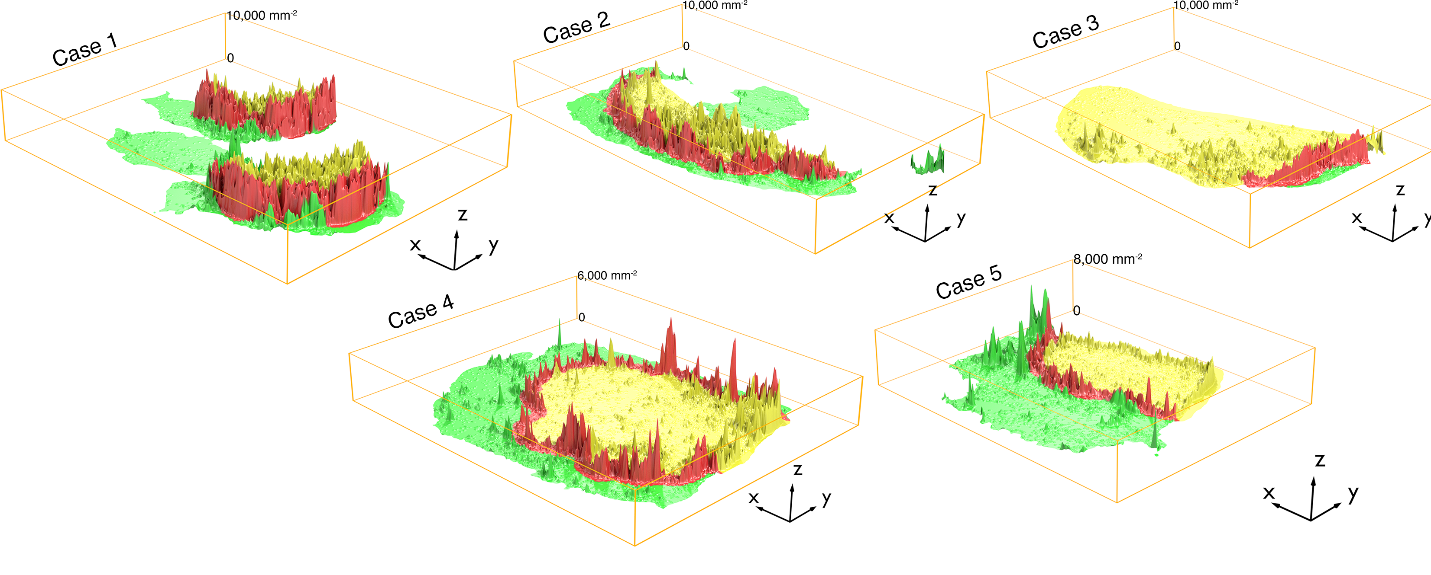
**

**Figure S9. 3D plots for CD8+ cells for Case 1-5.** Green: normal tissue (N); Red: invasive front (IF); Yellow: central tumor (CT).


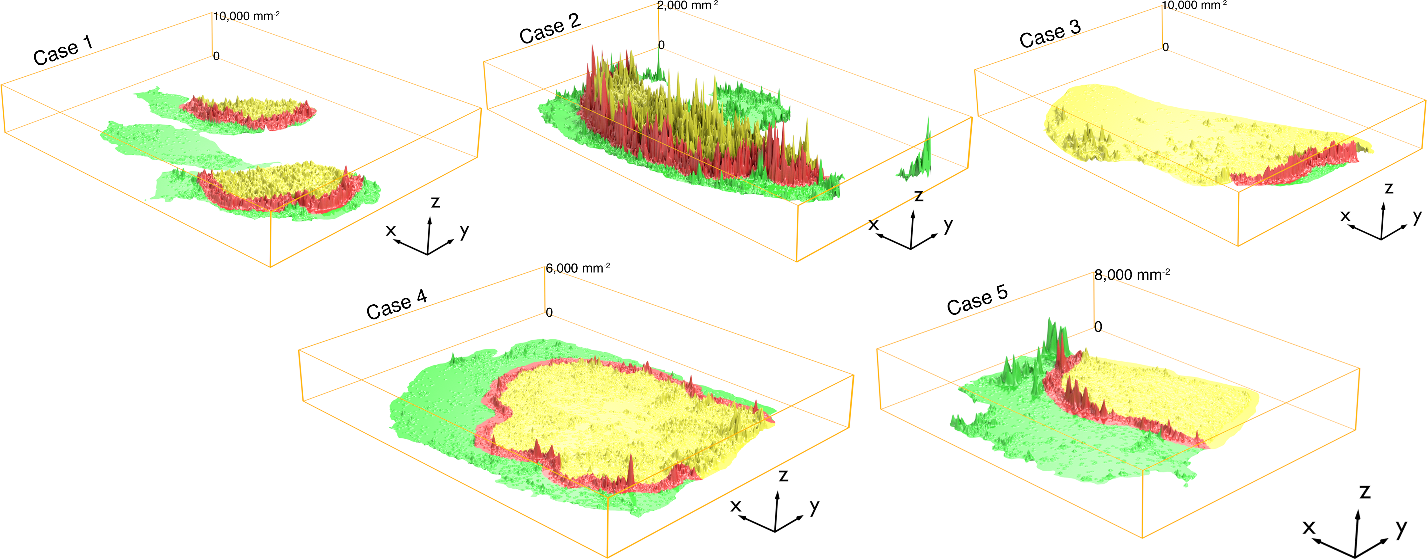


**Figure S10. 3D plots for FoxP3+ cells for Case 1-5.** Green: normal tissue (N); Red: invasive front (IF); Yellow: central tumor (CT).


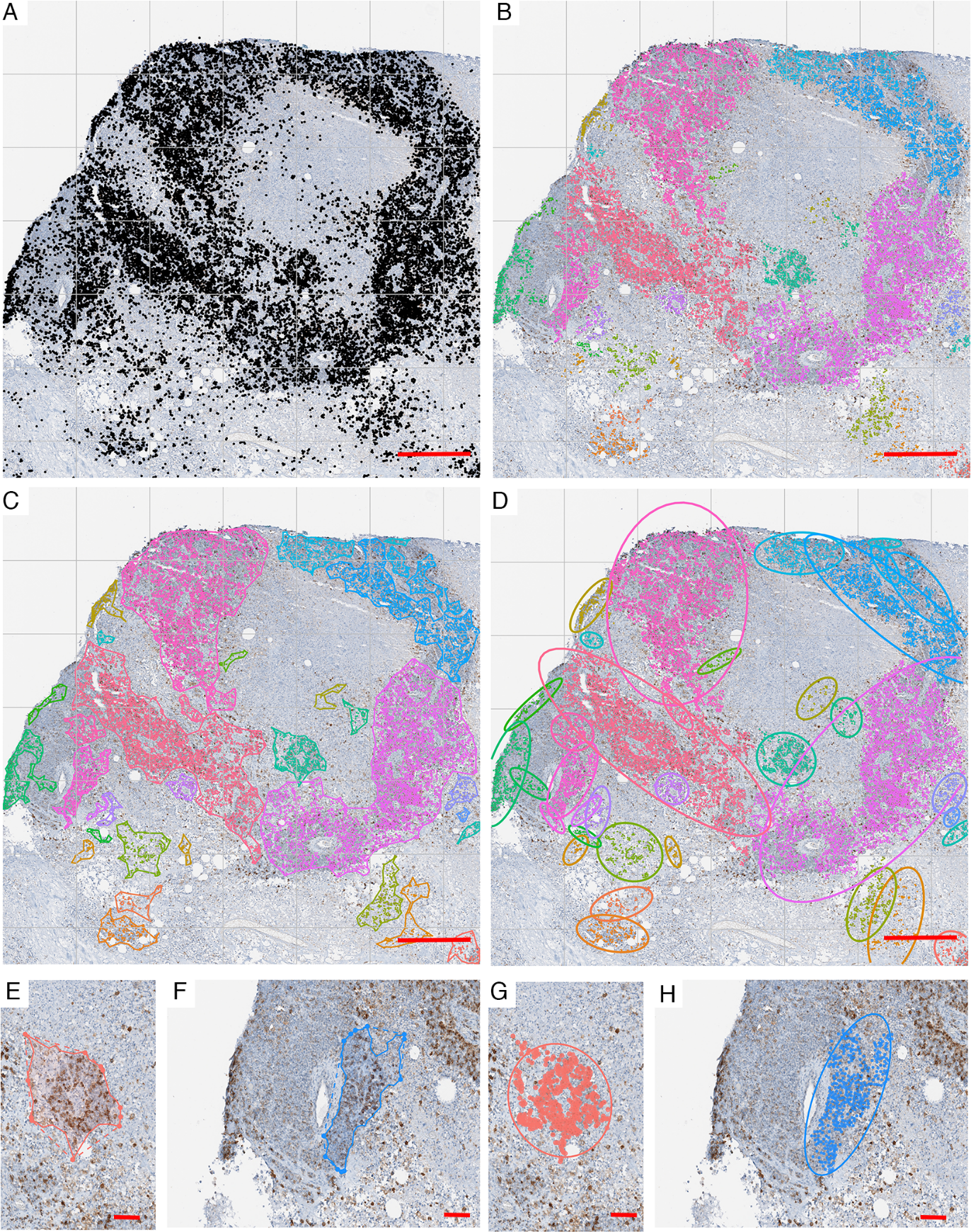


**Figure S11. Cluster analysis using shape descriptors.** **(A)** Point patterns of exemplar subregions. **(B)** Detected clusters using hierarchical-based algorithm. **(C)** For each cluster, we draw the alpha-shape, and **(D)** fitted ellipses. **(E)(F)** To calculate convexity and circularity, we further generate convex hull. **(G)(H)** The eccentricity of the same clusters is also calculated based on fitted ellipses. Scale: 0.5 mm (Panel A, B, C, D), 0.1 mm (Panel E, F, G, H).


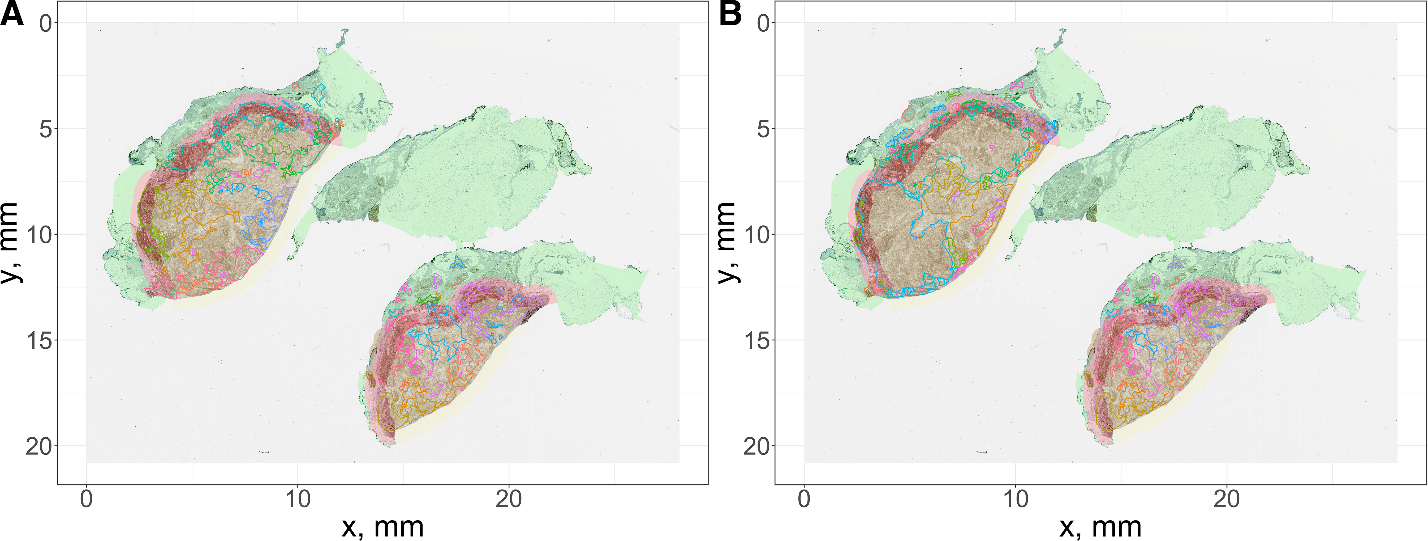


**Figure S12.­­_­­­_** **Results of CD8+ and FoxP3+ negative correlation analysis**. Green: normal tissue (N); Red: invasive front (IF); Yellow: central tumor (CT). Each cluster is represented by a colored outline. **(A)** Clusters of CD8+ cells with high DoC scores and FoxP3+ cells with low DoC scores for Case 1, AB. **(B)** Clusters of CD8+ cells with low DoC scores and FoxP3+ cells with high DoC scores for Case 1, AB.

**Table S1.** IHC segmentation and cell classification settings for QuPath.

| **Cell detection algorithm** | |
| --- | --- |
| **Setup parameters** | |
| Detection channel | Optical density sum |
| Background radius | 0.5µm |
| **Nucleus parameters** | |
| Background radius | 4µm |
| Median filter radius | 0µm |
| Sigma | 1.5µm |
| Minimum area | 10µm^2^ |
| Maximum area | 400µm^2^ |
| **Intensity parameters** | |
| Threshold | 0.02 |
| Max background intensity | 2 |
| Split by shape | Yes |
| Exclude DAB (membrane staining) | No |
| **Cell parameters** | |
| Cell expansion | 7.5µm |
| Include cell nucleus | Yes |
| **General parameters** | |
| Smooth boundaries | Yes |
| Make measurements | Yes |
| **Smooth object feature** | |
| Radius (FWHM) | 50µm |
| Smooth within classes | No |
| Use legacy feature names | No |
| **Cell classification algorithm** | |
| **Create region annotations** | |
| Width | 500 |
| Height | 500 |
| Size Unit | Pixels |
| Classification | Performance |
| Location | Random |
| **Train object classifier** | |
| Object filter | Detection (all) |
| Classifier type | Random trees (RTrees) |
| Features | All measurements |
| Classes | All classes |
| Training | Unlocked annotations |
| Advanced options | None |
| Live update | Yes |

**Table S2.** Feature list for Random Tree classifier.

| **Intensity features** | **Morphometry features** |  |
| --- | --- | --- |
| Nucleus: Hematoxylin OD mean | Nucleus: Area |  |
| Nucleus: Hematoxylin OD sum | Nucleus: Perimeter |  |
| Nucleus: Hematoxylin OD std dev | Nucleus: Circularity |  |
| Nucleus: Hematoxylin OD max | Nucleus: Max caliper |  |
| Nucleus: Hematoxylin OD min | Nucleus: Min Caliper |  |
| Nucleus: Hematoxylin OD range | Nucleus: Eccentricity |  |
| Nucleus: DAB OD mean | Cell: Area |  |
| Nucleus: DAB OD sum | Cell: Perimeter |  |
| Nucleus: DAB OD std dev | Cell: Circularity |  |
| Nucleus: DAB OD max | Cell: Max Caliper |  |
| Nucleus: DAB OD min | Cell: Min Caliper |  |
| Cell: Hematoxylin OD mean | Cell: Eccentricity |  |
| Cell: Hematoxylin OD sum | Nucleus/Cell area ratio |  |
| Cell: Hematoxylin OD std dev  Cell: Hematoxylin OD max  Cell: Hematoxylin OD min  Cell: DAB OD mean  Cell: DAB OD sum  Cell: DAB OD std dev  Cell: DAB OD max  Cell: DAB OD min  Cytoplasm: Hematoxylin OD mean  Cytoplasm: Hematoxylin OD sum  Cytoplasm: Hematoxylin OD std dev  Cytoplasm: Hematoxylin OD max  Cytoplasm: Hematoxylin OD min  Cytoplasm: DAB OD mean  Cytoplasm: DAB OD sum  Cytoplasm: DAB OD std dev  Cytoplasm: DAB OD max  Cytoplasm: DAB OD min | |  |
| **Smoothed features (all above, radius = 50µm)** | |  |

**Table S3**. Statistical summary for CD8+ and FoxP3+ immune markers correlation analysis (CD8+ dominate). N: normal tissue; IF: Invasive front; CT: central tumor.

| **Case** | **QDoC**  **(DoC Score)** | |  | **Correlated cell counts** | |  | **Percentage, %** | |  | **Cluster**  **density, mm^-2^** | | |
| --- | --- | --- | --- | --- | --- | --- | --- | --- | --- | --- | --- | --- |
|  | **CD8** | **FoxP3** |  | **CD8** | **FoxP3** |  | **CD8** | **FoxP3** |  | **N** | **IF** | **CT** |
| 1A | 0.60 | 0.67 |  | 47,005 | 41,097 |  | 19.3 | 44.5 |  | 0.11 | 1.59 | 1.37 |
| 1B | 0.28 | 0.37 |  | 35,920 | 23,994 |  | 26.9 | 35.8 |  | 0.27 | 1.65 | 1.70 |
| 2 | 0.39 | 0.46 |  | 26,486 | 43,721 |  | 17.1 | 50.5 |  | 0.09 | 2.11 | 0.83 |
| 3 | 0.27 | 0.22 |  | 15,264 | 20,245 |  | 25.9 | 43.6 |  | 0 | 1.71 | 0.37 |
| 4 | 0.59 | 0.89 |  | 10,108 | 34,791 |  | 11.9 | 60.0 |  | 0.06 | 1.13 | 0.20 |
| 5 | 0.97 | 0.82 |  | 4,281 | 19,661 |  | 9.0 | 62.0 |  | 0.19 | 0.95 | 0.14 |

**Table S4**. Statistical summary for CD8+ and FoxP3+ immune markers correlation analysis (FoxP3+ dominate). N: normal tissue; IF: Invasive front; CT: central tumor.

| **Case** | **QDoC**  **(DoC Score)** | |  | **Correlated cell counts** | |  | **Percentage, %** | |  | **Cluster**  **density, mm^-2^** | | |
| --- | --- | --- | --- | --- | --- | --- | --- | --- | --- | --- | --- | --- |
|  | **CD8** | **FoxP3** |  | **CD8** | **FoxP3** |  | **CD8** | **FoxP3** |  | **N** | **IF** | **CT** |
| 1A | 0.60 | 0.67 |  | 178,648 | 15,142 |  | 67.9 | 18.5 |  | 0.29 | 3.30 | 1.26 |
| 1B | 0.28 | 0.37 |  | 85,019 | 13,060 |  | 60.8 | 28.2 |  | 0.55 | 2.07 | 0.79 |
| 2 | 0.39 | 0.46 |  | 116,226 | 12,021 |  | 64.4 | 18.3 |  | 0.20 | 2.62 | 1.21 |
| 3 | 0.27 | 0.22 |  | 40,732 | 11,341 |  | 53.8 | 33.5 |  | 0 | 2.09 | 0.63 |
| 4 | 0.59 | 0.89 |  | 69,318 | 5,216 |  | 64.6 | 11.4 |  | 0.10 | 1.33 | 0.32 |
| 5 | 0.97 | 0.82 |  | 39,794 | 3,185 |  | 67.4 | 12.2 |  | 0.18 | 1.34 | 0.23 |

**Table S5**. Summary for image segmentation performance evaluation. TP: cells detected by both manual and algorithm approach; FP: cells detected by algorithm but rejected by manual approach; FN: cells detected by manual approach but are missed by the algorithm.

| **Case ID** | **Sample size** | **Manual count** | **Algorithm count** | **TP** | **FP** | **FN** |
| --- | --- | --- | --- | --- | --- | --- |
| **1A+1B** | 100 | 2,759 | 3,503 | 2,596 | 907 | 163 |
| **2** | 100 | 1,453 | 1,682 | 1,360 | 322 | 93 |
| **3** | 100 | 1,690 | 1,895 | 1,585 | 310 | 105 |
| **4** | 100 | 2,091 | 2,260 | 1,934 | 326 | 157 |
| **5** | 100 | 3,402 | 3,503 | 3,098 | 405 | 304 |

**Table S6**. Summary for total detected CD3+, CD4+, CD8+, CD20+, and FoxP3+ cells and region characterizations for all cases.

| **Case ID** | **Total CD3+ number** | **Total CD4+ number** | **Total CD8+ number** | **Total CD20+ number** | **Total FoxP3+ number** | **N area (mm^2^)** | **IF area**  **(mm^2^)** | **CT area**  **(mm^2^)** | **Total area**  **(mm^2^)** | **CD8+/FoxP3+ ratio** | **CD4+/FoxP3+ ratio** |
| --- | --- | --- | --- | --- | --- | --- | --- | --- | --- | --- | --- |
| **1A** | 261,308 | 424,807 | 225,653 | 186,837 | 56,239 | 27.9 | 17.0 | 47.5 | 92.4 | 4.0 | 7.6 |
| **1B** | 159,665 | 253,934 | 120,939 | 116,942 | 37,054 | 25.5 | 14.5 | 25.3 | 65.3 | 3.3 | 6.9 |
| **2** | 204,785 | 333,417 | 142,720 | 87,802 | 55,742 | 107.2 | 33.2 | 103.4 | 243.8 | 2.6 | 6.0 |
| **3** | 73,117 | 235,428 | 55,996 | 12,167 | 31,586 | 4.6 | 10.5 | 238.3 | 253.4 | 1.8 | 7.5 |
| **4** | 44,018 | 42,277 | 44,885 | 8,800 | 23,728 | 166.6 | 56.4 | 244.3 | 467.3 | 1.9 | 1.8 |
| **5** | 86,298 | 195,930 | 79,426 | 16,539 | 40,007 | 113.7 | 17.8 | 90.6 | 222.1 | 2.0 | 4.9 |

**References**

Borovec, J., Munoz-Barrutia, A., and Kybic, J. (2018). Benchmarking of image registration methods for differently stained histological slides. *2018 25th IEEE International Conference on Image Processing (ICIP)***,** 3368-3372. doi: 10.1109/ICIP.2018.8451040.

Cimino-Mathews, A., Thompson, E., Taube, J.M., Ye, X., Lu, Y., Meeker, A., et al. (2016). PD-L1 (B7-H1) expression and the immune tumor microenvironment in primary and metastatic breast carcinomas. *Human pathology* 47**,** 52-63. doi: 10.1016/j.humpath.2015.09.003.

De Chaumont, F., Dallongeville, S., Chenouard, N., Hervé, N., Pop, S., Provoost, T., et al. (2012). Icy: an open bioimage informatics platform for extended reproducible research. *Nature methods* 9**,** 690. doi: 10.1038/nmeth.2075.

Qidwai, U., and Chen, C.-H. (2009). *Digital image processing: an algorithmic approach with MATLAB.* Chapman and Hall/CRC.

Team, R.C. (2015). *R package stats: A language and environment for statistical computing. R Package Version 0.1.0.* Available online at: <https://cran.r-project.org/web/packages/STAT/index.html>.

Wang, C.-W., Ka, S.-M., and Chen, A. (2014). Robust image registration of biological microscopic images. *Scientific Reports* 4**,** 6050. doi: 10.1038/srep06050.
